# Change in exercise capacity, physical activity and motivation for physical activity at 12 months after a cardiac rehabilitation program in coronary heart disease patients: a prospective, monocentric and observational study

**DOI:** 10.7717/peerj.18885

**Published:** 2025-02-14

**Authors:** Paul Da Ros Vettoretto, Anne-Armelle Bouffart, Youna Gourronc, Anne-Charlotte Baron, Marie Gaume, Florian Congnard, Bénédicte Noury-Desvaux, Pierre-Yves de Müllenheim

**Affiliations:** 1 Cardiac Rehabilitation Unit, Hospital Center of Cholet, Cholet, France; 2 Clinical Research Unit, Hospital Center of Cholet, Cholet, France; 3 APCoSS, UCO-IFEPSA, Les Ponts-de-Cé, France

**Keywords:** Coronary artery disease, Cardiac rehabilitation program, Walking capacity, Movement behaviours, Self-determined motivation, Follow-up

## Abstract

**Background:**

Exercise capacity (EC) and physical activity (PA) are relevant predictors of mortality in patients with coronary heart disease (CHD) but the CHD-specific long-term trajectories of these outcomes after a cardiac rehabilitation (CR) program are not well known. The main objective of this study was to determine the mean change in EC (6-min walking test (6MWT) distance) in CHD patients at 12 months after a CR program compared to the end of the program. We also performed a series of exploratory analyses: (i) estimating the decile shifts and the typical (median) individual change for EC, PA (International Physical Activity Questionnaire-Short Form Metabolic Equivalent of Task (IPAQ-SF MET)-min/week), and motivation for PA (Echelle de Motivation envers l’Activité Physique en contexte de Santé (EMAPS) scores) over the 12-month follow-up period; (ii) characterizing the PA motivational profiles at the end of the program and 12 months after the program; (iii) characterizing the barriers to PA perceived at 12 months; and (iv) estimating the categories of changes in EC and PA over time and their potential predictors.

**Methods:**

Eighty-three patients were recruited at the end of a CR program.

**Results:**

For an average patient, EC was trivially increased at 12 months. However, the decile shifts analysis did not confirm that the positive shift of the distribution of the performances over time was uniform. In contrast, we observed a significant decrease in PA between the end of the program and 12 months post-program but not between 6 and 12 months post-program when considering both the group of patients as a whole and the typical individual change. The results regarding motivation for PA were mixed, with significant and non-uniform shifts of the deciles towards scores depicting degrees of autonomous and controlled motivations as well as amotivation that would be more in favor of PA, but with no significant typical individual changes except for introjected regulation. Two motivational profiles were identified both at the end of the program and 12 months after the program: one with a very high level of autonomous motivation and a high level of introjected regulation; and another one with a high level of autonomous motivation and a moderate level of introjected regulation. Unfavorable weather, lack of time, fatigue, and fear of injury were the main barriers to PA at 12 months post-program. The change in EC and PA could be categorized into different classes without the possibility to determine any potential predictor of the assignment to a given class. Overall, these results suggest that clinicians managing a CR program with CHD patients as the one implemented in the present study may expect slightly positive or at least steady trajectories in EC, PA (after 6 months), and motivation for PA during the year after the program when considering the bulks of the distributions of patient scores. However, these global trajectories are actually the results of heterogeneous individual changes with some profiles of patients who could need a particular attention.

## Introduction

Coronary heart disease (CHD) is one of the major clinical heart and circulatory disease conditions, with an estimate of more than 244 million of people suffering from ischemic heart disease worldwide (Tsao et al., 2023). In CHD patients, mortality risk is negatively associated with exercise capacity (*i.e*., cardiorespiratory fitness) as assessed (directly measured or estimated) using cardiopulmonary (Ezzatvar et al., 2021) and walking distance tests (Cacciatore et al., 2012) and is also negatively related to self-reported physical activity level (Bouisset et al., 2020). As a result, cardiac rehabilitation (CR) programs, which include exercise training and physical activity counselling as core components (Balady et al., 2007), are recommended (Virani et al., 2023) for CHD patients with appropriate indications (*i.e*., recent myocardial infarction, percutaneous coronary intervention, or coronary artery bypass grafting; stable angina or recent heart transplant; recent spontaneous coronary artery dissection event).

While progress in exercise capacity is beneficial for survival in CHD patients, it has been recently shown that exercise capacity reached at the end of a CR program would be the best predictor for long-term survival in CHD patients compared to baseline and change in exercise capacity during the CR program (Carbone et al., 2022). Moreover, beyond the physical activity level (*i.e*., the volume or dose of physical activity performed over a typical day or week) observed at a given time point, physical activity trajectory would have a significant influence on mortality in CHD patients (Gonzalez-Jaramillo et al., 2022), being active and remaining active over time being the most favourable trajectory compared to being inactive and remaining inactive. These recent results support the interest of investigating the long-term evolution of exercise capacity and physical activity level in CHD patients after a CR program. Such an investigation could also be coupled with the characterization of the individual determinants (*e.g*., motivations, self-efficacy, exercise history, skills, and other health behaviours) and environmental determinants (*e.g*., access, cost, and time barriers and social and cultural supports) of physical activity behaviour (Sherwood & Jeffery, 2000). This could help to understand the reasons of a possible change in physical activity and hence exercise capacity over time in cardiac patients. In particular, analysing motivation for physical activity could be here an appropriate approach supported by the well-established self-determination theory framework (Ryan et al., 2009) stating that the adoption and maintenance of physical activity behaviour is dependent on the form of motivation (autonomous *vs*. controlled) and/or on regulations (integrated, identified, introjected, external) related to physical activity, with a particular positive influence of autonomous (identified and intrinsic) regulations (Teixeira et al., 2012). This statement is based on both cross-sectional and longitudinal (observational and experimental) studies (Teixeira et al., 2012), including studies conducted in cardiac patients after a CR program (Russell & Bray, 2009).

Several studies investigated the evolution of exercise capacity and physical activity level after a CR program (Boesch et al., 2005; Giallauria et al., 2006; Mildestvedt, Meland & Eide, 2008; Steinacker et al., 2011; Pavy, Tisseau & Caillon, 2011; Ramadi et al., 2016; Nilsson et al., 2018; Racodon, Porrovecchio & Pezé, 2019; Taylor et al., 2020; Kim et al., 2021a; Batalik et al., 2021), but not all tested the change between the end of the CR program and the follow-up measurements (Boesch et al., 2005; Giallauria et al., 2006; Pavy, Tisseau & Caillon, 2011; Ramadi et al., 2016; Nilsson et al., 2018). Most of these last studies found no change in exercise capacity as assessed using either a maximal treadmill/cycling exercise test or the six-minute walking test (Boesch et al., 2005; Pavy, Tisseau & Caillon, 2011; Ramadi et al., 2016; Nilsson et al., 2018) and they found no change in physical activity using a questionnaire (Ramadi et al., 2016; Nilsson et al., 2018). However, very few studies actually tested a change after a CR program with ≥1-year follow-up measurements (Boesch et al., 2005; Nilsson et al., 2018). Moreover, to our knowledge, only one study analysed the natural change in motivation for physical activity over time in cardiac patients after a CR program, with the finding of a decline in the Physical Activity and Leisure Motivation Scale during the 9 months after the program (Kim et al., 2021b). Finally, no studies implemented analyses to quantify, beyond the change in the central tendency (*e.g*., the mean), how the whole distribution of patients’ results (exercise capacity, physical activity level, or motivation for physical activity) evolves over time after a CR program, as well as the typical (median) individual change, letting unclear the real success of the program for maintaining or increasing exercise capacity, physical activity, and motivation for physical activity over time when considering the whole cohort of the patients, not the average patient only.

The main objective of the present study was to determine if there is a change in mean exercise capacity of CHD patients 12 months after completing a CR program compared to the end of the program, with no consideration of the results at the entry in the program. As we expected patients adopt a physically active lifestyle after a CR program without looking for a progression in the dose of physical exercises over time, we hypothesized there is no change in exercise capacity at 12 months after the program. A first secondary objective was to explore how the distributions of exercise capacity, physical activity level and motivation for physical activity evolve between the end of the CR program and 12 months after the program, as well as the typical individual changes in these outcomes over this period of time. As these last analyses were exploratory, we had no particular rationale to set specific hypotheses and, therefore, the classically used hypothesis of the absence of change was again considered here. Other secondary objectives were (i) to describe the change in the motivational profile for physical activity using a clustering approach, (ii) to describe barriers to physical activity reported by CHD patients 12 months after the CR program, and (iii) to explore the potential classes of change over time in exercise capacity and physical activity and their respective predictors. A preprint version of this article has been peer-reviewed and recommended by PCI Health & Mov Sci (Impellizzeri, 2024).

## Materials and Methods

### Study overview

We conducted a prospective, monocentric, and observational study coupled with a convenience sampling method. This study has been approved by the relevant regulatory authorities and institutional ethics committee (“APA&Co” project; CPP Ile de France X, France; CNRIPH reference: 20.12.10.68140/Id. 10644; ClinicalTrials.gov ID: NCT04732923). The recruitment of the participants was conducted at the hospital center of Cholet (France). It consisted of orally presenting, by the same person (a teacher of adapted physical activity), the study at the beginning of the last week of the CR program (week 4) to the wave of patients currently enrolled in the CR program and who fulfilled the following criteria: has an acute coronary syndrome; is ≤70 years old; has completed the CR program; can respect the protocol constraints; is registered with, or can benefit from, social security; has signed the informed consent form; has not a heart failure condition; is not a pregnant, parturient, or breast feeding woman; is not deprived of liberty due to judicial or administrative decision; is not forced to follow treatments for psychiatric disorders; is not hosted in a medical and social establishment for reasons other than research; is not concerned by legal safeguards or can express his/her consent. Patients could then notice to the investigators their interest to participate to the study during the visit planned at the end of the last week of the CR program. The recruitment and data collection periods took near of 2 years (start of the study: 2021-02-10; completion of the study: 2023-02-09).

We obtained information regarding age, sex, height, and surgery history at the end of the CR program. Participants also completed the measurements of weight, exercise capacity, physical activity level, and motivation for physical activity at three time points: at the end of the CR program (last week of the CR program), at 6 months after the CR program, and at 12 months after the CR program. Barriers to physical activity were assessed at 12 months after the CR program only.

### Content of the cardiac rehabilitation program

The CR program used in the present study was based on the guidelines from the French Society of Cardiology (Pavy et al., 2011). Patients who followed the program had to come to the center four days (08:30 am to 4:30 pm) per week during four consecutive weeks. Patients could have five collective activities per day. A typical week included the following sessions: four indoor (leg cycling) aerobic training sessions (content: 40 min with both continuous and intermittent exercises; intensity based on the training heart rate determined by the cardiologist following the exercise test at the entry in the program); two to three sessions of resistance training (content: 45 min (10 exercises related to the main muscle groups), three series of 10 repetitions at 40% of 1 RM per exercise); two to three sessions of gymnastic (content: 60 min exercising the main muscle groups using body weight or small weights (*e.g*., swiss ball, medicine balls, barbells)); two to three sessions of adapted physical activity (content: 60 min where various activities can be performed such as table tennis, badminton, blowgun, archery, volleyball, hockey, *etc*.); two to three sessions of outdoor walking or Nordic walking (content: 30 to 60 min at free pace following marked footpaths); two to three sessions of lifestyle counselling; an individual consultation depending on the needs of the patient (with either a cardiologist, a psychologist, a dietician or a tobaccologist); and an ergotherapy session for some patients. Finally, a consultation at 6 months after the end of the program was planned with a nurse. During this consultation, the nurse conducted an interview-based assessment of patient’s knowledge about the disease, the treatments, the strategies to cope with stress, the benefits and risks of physical activities, and the principles of a healthy diet. They also assessed quality of life, and asked the patients about the return to work. We took the opportunity of this follow-up visit to perform intermediary evaluations (exercise capacity, physical activity level, motivation for physical activity). These evaluations were performed following the consultation with the nurse and were judged useful for monitoring the patients and for conducting potential secondary analyses of the project. During this visit, patients were not informed on the results obtained following their previous evaluations, and they did not receive any pieces of advice related to these intermediary evaluations.

During the program, all participants were treated with β-blockers, antiplatelet agents, statins and angiotensin-converting enzyme inhibitors.

### Tests and instruments

#### Exercise capacity

Exercise capacity was studied throughout the use of the distance performed during the 6-min walking test (6MWT). All 6MWTs were conducted in a 25-m corridor by the same assessor. At each test, the principle of the test was explained to the participant, and the same information was provided during the test, that is, the time elapsed at each minute of the test. Of note, in CHD patients, the 6MWT has a 2% to 8% test-retest mean change with an intraclass correlation coefficient (ICC) of 0.97, as well as a moderate correlation (around 0.5) with peak oxygen uptake (Bellet, Adams & Morris, 2012). Because the test performed at the end of the CR program in the present study actually was the second test experienced by the participants after the first one performed at the beginning of the CR program, the potential learning effect may have been decreased for the follow-up measurements.

#### Physical activity level

Physical activity level was assessed using the International Physical Activity Questionnaire-Short Form (IPAQ-SF) and was expressed in metabolic equivalent (MET)-min/week. For the first assessment, participants were asked to consider physical activity performed both at the hospital center where the CR program was completed and outside the hospital. The IPAQ-SF measures the number of days and the usual daily time spent during the previous week in (i) moderate physical activity (MPA), (ii) vigorous physical activity (VPA), (iii) walking, and (iv) sitting, but only considering bouts longer than 10 min (except for sitting). Then, it allows the computation of weekly physical activity dose in MET-min by multiplying weekly durations of physical activity by appropriate coefficients (4.0 for MPA, 8.0 for VPA and 3.3 for walking). While the IPAQ-SF has a weak validity for measuring physical activity dose, with correlations with objective standards ranging from 0.09 to 0.39 (Lee et al., 2011), it has an acceptable test-retest reliability with a pooled Spearman coefficient based on the literature of 0.76 (Craig et al., 2003). For the present study, we used the French IPAQ-SF version proposed in the ‘Pralimap’ trial (Pralimap, 2007) shown in Supplemental Material 1. With this questionnaire, total physical activity durations were directly recalled at the week level, not using a typical daily duration as in the original English version. Furthermore, total walking activity had to be reported using the number of ≥10-min periods (consecutive or not) performed during the week. This number was then multiplied by 10 to obtain total weekly walking minutes. To the best of our knowledge, the psychometric properties of this specific IPAQ-SF version are unclear.

#### Motivation for physical activity

Motivation for physical activity was measured using the EMAPS (Echelle de Motivation envers l’Activité Physique en contexte de Santé) questionnaire (Boiché et al., 2019) with permission to use this instrument from the copyright holders. The EMAPS is based on the self-determination theory framework and uses 18 items, with three items for each of the six motivational constructs of the theoretical framework: intrinsic motivation (“individuals perform a behaviour for the pleasure and satisfaction they directly derive from it”, p.1), integrated regulation (“the behaviour is conceived as congruent with individuals’ core values and personal lifestyle”, p.2), identified regulation (“individuals perform an action because they believe this could help them to reach a personally valued goal”, p.2), introjected regulation (“pressures to execute the behaviour have been interiorized, and (…) individuals act to avoid negative feelings of shame or guilt, or to enhance feelings of self-worth”, p.2), external regulation (“the behaviour is performed in response to external contingencies such as rewards or punishments, and (…) individuals comply to social pressure”, p.2), and amotivation (“individuals feel that there is no way to achieve positive outcomes through their actions”, p.1) (Boiché et al., 2019). Each item was scored using a seven-point Likert scale (1 = “Does not correspond at all”; 7 = “Corresponds very strongly”). Then, a final score for each of the motivational constructs was obtained by averaging the scores related to the three corresponding items. For one patient, a score related to one item was lacking. The average score for the corresponding motivational construct was then computed from the two remaining items. The EMAPS, which is a French language questionnaire, is a valid tool with reliability ICCs ranging from 0.616 to 0.790 depending on the motivational construct (Boiché et al., 2019). In the present article, an increase in the scores depicting the most autonomous forms of motivation (intrinsic motivation, integrated regulation, identified regulation) and introjected regulation on one side, and a decrease in the scores depicting external regulation and amotivation on another side, were considered as beneficial. These considerations are based on the work by Teixeira et al. (2012) who found positive associations of autonomous forms of motivation and introjected regulation with physical activity, and negative associations of external regulation and amotivation with physical activity.

#### Barriers to physical activity

Barriers to physical activity were assessed using the items proposed by the French National Authority for Health (Haute Autorité de Santé, 2022): unfavourable weather; lack of time; heavy effort/too tired; fear of injury/pain; lack of interest; difficulty to move; too old; social isolation/weak social network; too costly. For each of the items, the participant had to indicate whether the item was considered as a barrier or not.

### Sample size determination

Sample size was determined from the objective of analysing the change in mean 6MWT distance at 12 months post-program compared to the end of a CR program in CHD patients. More precisely, sample size determination was based on the results of a French study by Pavy et al. (2011) who found, in 202 patients with CHD, a mean ± SD of 511 ± 91 m for the 6MWT distance at the end of the CR program. Assuming the participants of the present study would achieve approximately the same average exercise capacity at the end of the CR program as in the Pavy et al. (2011) study, we considered that an absolute mean change ≥50 m post-program could be viewed as important since it would represent a change ≥~10% of the mean 6MWT distance achieved at the end of the program, that is a change greater than the potential learning effect of 2% to 8% (Bellet, Adams & Morris, 2012) in the case of a positive change. Sample size was then computed to be able to detect, if any, effect sizes compatible with such a population mean change (±50 m), assuming an alpha level of 5% and a power of 90%. To determine sample size, the considered mean change of 50 m described above had to be converted to a standardized effect size (d_z_) by dividing this value of 50 m by the expected within-subject SD of differences. As we had no information about the expected within-subject SD of differences, we first decided to conduct a power analysis for the study as if it were a between-subject design using a theoretical population Cohen’s d_s_ effect size computed from a mean difference of 50 m for the numerator and an assumed pooled SD of 91 m for the denominator basing on the Pavy et al. (2011) results. This led to a d_s_ of ≈0.55. Then, we computed the sample size theoretically required (NB) to detect a statistically significant effect, assuming a 90% power, an alpha level of 5%, and a true d_s_ of 0.55, while using a two-sided unpaired t-test (NB = 140). Finally, we derived from NB the sample size for a within-subject design (NW) based on the formula NW = NB×(1−r)/2 provided by Maxwell, Delaney & Kelley (2004) cited by Caldwell et al. (2022), where r is the correlation between the two dependent variables in a within-subject design study. Here, r was set to 0 to likely overestimate rather than underestimate the sample size required to detect a statistically significant effect assuming a true d_s_ of 0.55. The final sample size obtained was 70. Assuming a 10% loss of participants during the study, we aimed at recruiting at least 77 participants. Power calculation was performed using the BiostaTGV software (Sentinelles, 2021). Supplemental Material 2 shows how the software was used to determine the required sample size.

### Statistical analysis

All data analyses were performed using R programming language v4.3.2. (R Core Team, 2023) and the RStudio environment ("Chocolate Cosmos", v2024.04.1).

#### Participant characteristics

The distributions of the continuous variables were graphically analysed using raincloud plots and q-q plots. In the tables, the continuous variables are shown as means ± SD when the distributions appeared approximately Gaussian or as medians and interquartile ranges (IQR, 25^th^ percentile–75^th^ percentile) otherwise. The categorical variables have been summarized as counts and percentages.

#### Change in 6MWT distance, IPAQ-SF MET-min/week and EMAPS scores

Only participants who had data at both the time points of interest were used for analysing the considered variable. First, as initially planned during the power analysis stage of the study, we tested a null difference between the means of the 6MWT distances obtained respectively at 12 months after the CR program and at the end of the program using a two-sided paired t-test. The results of the t-test have been reported along with the Pearson correlation depicting the strength of the linear link between the 6MWT performances at 0 and 12 months post-program, and with a Cohen’s d effect size using the average standard deviation (d_av_). Then, for 6MWT distance, International Physical Activity Questionnaire-Short Form Metabolic Equivalent of Task (IPAQ-SF-MET)-min/week and EMAPS scores, we exploratory analysed (i) the change in the distribution of the scores (no consideration of the individual trajectories) and (ii) the distribution of the participant changes (consideration of the individual trajectories) between the two time points of interest (0 and 12 months for 6MWT, IPAQ-SF and EMAPS; and 6 months and 12 months for IPAQ-SF only to also consider the change in “true” free-living physical activity behaviour as the measurement at 0 month for the IPAQ-SF was not related to a week after patient discharge and thus did not reflect the participant natural behaviour; thus, the change between 6 and 12 months in IPAQ-SF MET-min/week was the main analysis of physical activity in the present study). These analyses were conducted respectively using (i) a shift function and (ii) the median of the changes and a difference asymmetry function. Of note, a shift function consists of plotting and allowing inferences about the differences between the quantiles of two distributions (*e.g*., quantile 0.10 of 6MWT distance at 12 months post-program minus quantile 0.10 of 6MWT distance at the end of the program) as a function of the quantiles of a reference distribution (*e.g*., the quantiles of 6MWT distance at the end of the program). In the present article, the shift functions use the differences between the estimated deciles of the two marginal distributions to be compared. A flat shift function above or below 0 will reveal respectively a uniform positive or negative shift of the distribution (same distribution shape, but shifted position), while an increasing or a decreasing shift function will reveal a more positive shift in favour of the last or the first deciles of the distribution, respectively. Regarding the difference asymmetry function, it shows the sum of quantiles = q + (1−q) of the distribution of the individual differences between the two time points of interest, with 0.05 standing for the sum of quantile 0.05 + quantile 0.95, 0.10 standing for the sum of quantile 0.10 + quantile 0.90, *etc*., (Rousselet, Pernet & Wilcox, 2017). A flat, increasing, or decreasing difference asymmetry function will depict a symmetric, left-skewed, or right-skewed distribution of the individual differences, respectively, and the null, positive, or negative parts of the function will highlight respectively a rather neutral, positive, or negative shift of the participant scores. The estimates of the deciles of the marginal distributions, the quantiles (including the median) of the individual changes, the decile shifts (shift function) and the quantile sums (difference asymmetry function) shown in the present article were obtained using the Harrell-Davis estimator (Rousselet, Pernet & Wilcox, 2017). The medians of the individual changes, the decile differences from the shift functions and the quantile sums from the difference asymmetry functions are provided with related percentile bootstrap 95% confidence intervals (non-adjusted for multiple comparisons) and also, only for the shift and difference asymmetry functions, *P* values adjusted for multiple comparisons. *P* values were adjusted using the Benjamini-Hochberg False Discovery Rate method (Yekutieli & Benjamini, 1999). Claims about changes were made based on the *P* values for the t-test and the shift and difference asymmetry functions, and on the 95% confidence interval (95% CI) for the median of the differences, this assuming a 5% error rate in the long run.

#### Change in the profile of motivation for physical activity

The change in motivation for physical activity over time was also explored using the change in the motivational profile analysed using a clustering approach. The rationale for this analysis was that a single form of motivation may not capture the actual complexity of a motivational profile, as in people who would present both high levels of autonomous and controlled motivation for example (Gillet, Vallerand & Rosnet, 2009; Gillet, Berjot & Paty, 2010; Friederichs et al., 2015). To perform such an analysis at each of the time points of interest (0 and 12 months post-CR program), we first scaled the motivation variables (EMAPS scores). Then, we confirmed the possibility to conduct a clustering analysis both graphically (using a dissimilarity matrix image) and statistically (Hopkins statistic > 0.7) (Kassambara, 2017). Then, we implemented a k-medoid approach to partition the considered EMAPS scores dataset into k groups or clusters, each cluster being represented by a participant in the cluster (*i.e*., the medoid). This approach was deemed more appropriate than the classically used k-means approach (Gillet, Vallerand & Rosnet, 2009; Gillet, Berjot & Paty, 2010; Friederichs et al., 2015) as our datasets presented several multivariate outliers as assessed using the minimum covariance determinant (Thériault et al., 2024), and the k-medoid approach is less sensitive to outliers (Kassambara, 2017). More precisely, we used the Partitioning Around Medoids (PAM) algorithm that aimed at building k clusters so that the global average sum of the intra-cluster dissimilarities (based on the Euclidean distances from the medoid) was minimal. The optimal number of clusters k to be built by the algorithm was determined using the average silhouette method that measures the quality of a clustering: the higher the value of the average silhouette, the better the clustering (*i.e*., higher intra-cluster similarities, higher inter-cluster dissimilarities). The clusters we obtained were named using a similar approach as in Gillet, Vallerand & Rosnet (2009) consisting of introducing, in the name of the cluster, information related to the motivational concepts and the magnitude of the motivational scores that highlighted the between-cluster differences. The pertinence of the clusters we obtained was also assessed by comparing their respective EMAPS scores using nonparametric multivariate analysis (Burchett et al., 2017), both at 0 month and at 12 months post-CR program. We considered that the participants related to the identified clusters came from different multivariate distributions when the *P* value associated to the ANOVA type statistic (F-approximation) was below or equal to 0.05. When there was an overall difference between the compared clusters, multiple comparisons were performed to determine for which EMAPS scores and combinations of EMAPS scores there were differences between the clusters, again using the ANOVA type statistic (F-approximation) while maintaining the overall Type I error rate at 5%. The proportions of participants related to a given scenario of profile transition between 0 and 12 months post-CR program were computed along with their respective 95% Clopper and Pearson CIs.

#### Barriers to physical activity

Proportions of the participants citing a given barrier to physical activity at 12 months after the CR program were computed along with their respective 95% Clopper and Pearson CIs.

#### Predictors of the change in 6MWT distance and IPAQ-SF MET-min/week

We finally explored the possibility to identify predictors of the change in 6MWT (0–12 months) distance and IPAQ-SF MET-min/week (6–12 months) using a latent class trajectory modelling approach (Proust-Lima, Philipps & Liquet, 2017). This approach consisted first of building an initial linear mixed model with the outcome of interest as response variable and the month of measurement as explanatory variable and a random intercept on the participants. Then, a series of models were derived from this initial model and tested to determine whether the change in the outcome of interest over the follow-up period could be categorized into different classes depending on the direction and the magnitude of the change. These new models were latent class mixed models as they included a term allowing the determination of a class-specific fixed effect (here the month of measurement) in the mixed model (Proust-Lima, Philipps & Liquet, 2017). Four groups of latent class linear mixed models were tested for the possibility to have two to five classes of change in the outcome of interest as previously proposed (Limpens et al., 2023). As these models needed initial values to allow the model building algorithm to converge, we implemented, as recommended (Proust-Lima, Philipps & Liquet, 2017), a grid search strategy. In the present analysis, this consisted of testing, for a given number of expected latent classes, 100 models respectively based on 100 random vectors of initial values obtained from the parameters defined with the initial linear mixed model (1 class). The best model for a given number of classes obtained from the grid search strategy was then retained for subsequently comparing the different models related to the different numbers of latent classes. For a given outcome of interest, the most appropriate latent class mixed model (*i.e*., the most appropriate number of classes) was chosen based on a combination of the Akaïke Information Criterion (AIC), the Bayesian Information Criterion (BIC), entropy, the numbers of study participants posteriorly assigned to a given class by the model considered (Proust-Lima, Philipps & Liquet, 2017), and also the possibility to have a model able to converge during the subsequent analysis of the predictors of the latent classes. The analysis of potential predictors of the assignment to a given class was performed using a multinomial regression model (with the class showing the best mean change as reference class) with the following potential predictors: 6-min walking distance, physical activity level and motivational profile at the end of the program; and the two main barriers to physical activity (unfavourable weather and lack of time). The inference technique related to this analysis was a 2-stage estimation of the joint likelihood of the primary (final) latent class mixed model and the secondary regression. Covariates in the latent class mixed models and the multinomial regression models were considered as statistically significant when the *P* values associated to the corresponding Wald tests were ≤ 0.05 (Proust-Lima, Philipps & Liquet, 2017).

## Results

A total of 83 participants were included in the study, with 10 out of 93 patients (11%) who refused to participate. The participants enrolled in the CR program on average 17 ± 2.5 weeks after their first cardiac event (minimum: 4 weeks; maximum: 46 weeks, which is a very high number concerning only one patient due to planning difficulties). Participant characteristics are shown in Table 1. Participants were on average overweight, mainly men, most of them having had an angioplasty. Two women were on hormone therapy to control hypothyroidism. Information regarding the treatments taken by the participants during the follow-up period of the study was available only for those who were medically followed at the hospital center of Cholet (*N* = 53). This information was obtained at 9.4 ± 2.7 months post-CR program during a follow-up visit that was not a part of the study. At this visit, the distribution of the treatments was the following: 87% with β-blockers, 96% with antiplatelet agents, 96% with statins and 53% with angiotensin-converting enzyme inhibitors.

**Table 1:** Participant characteristics at the inclusion stage (*N* = 83).

Participant characteristic	Descriptive statistics
Sex (W/M)	6/77 (7%/93%)
Age (yr)	59 (53–64)	
Height (cm)	173 ± 7
Weight (kg)	82.5 ± 13.6
Body mass index (kg/m^2^)	27.6 ± 4.0
Surgery history		
Angioplasty	66 (79.5%)	
Bypass	21 (25.3%)

**Note:**

W, women; M, men. Statistics are counts (%) for categorical variables and mean ± SD or median (25^th^–75^th^ percentiles) for numeric variables.

**Figure 1:**
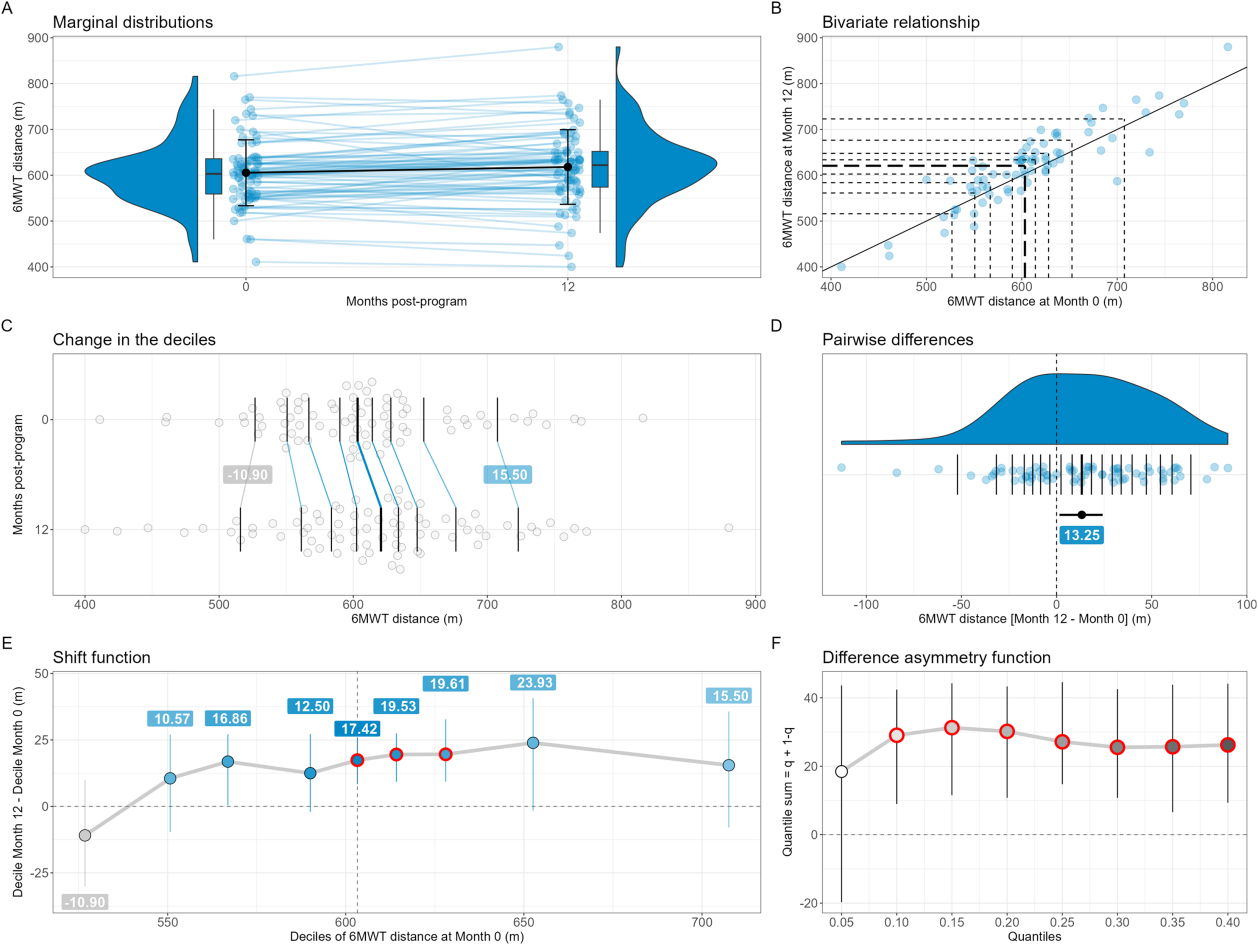
Change in 6-min walking test (6MWT) distance between 0 and 12 months (*N* = 75). On (A), the errors bars over the points are the standard deviations around the means, while on (D) it is the percentile bootstrap 95% confidence interval around the median estimate. On (E) and (F), the error bars are percentile bootstrap 95% confidence intervals not corrected for multiple comparisons. On (B), (C) and (D), the horizontal and/or vertical segments are the estimates of the deciles (B and C) or the quantiles (D, step of 0.05) of the distributions; the thickest segments are the median estimates. On (B), the diagonal black line depicts the identity line. If any, significant results (based on adjusted *P* values) in the shift (E) and difference asymmetry (F) functions are highlighted using thick red circles. A small pseudo-random movement has been added horizontally and vertically to the raw data displayed on (B) to minimize the presence of points fully overlapped. The estimates of the deciles of the marginal distributions (B, C and D), the quantiles of the individual differences (D), the decile differences (E) and the quantile sums (F) have been computed using the Harrell-Davis estimator.

**Figure 2:**
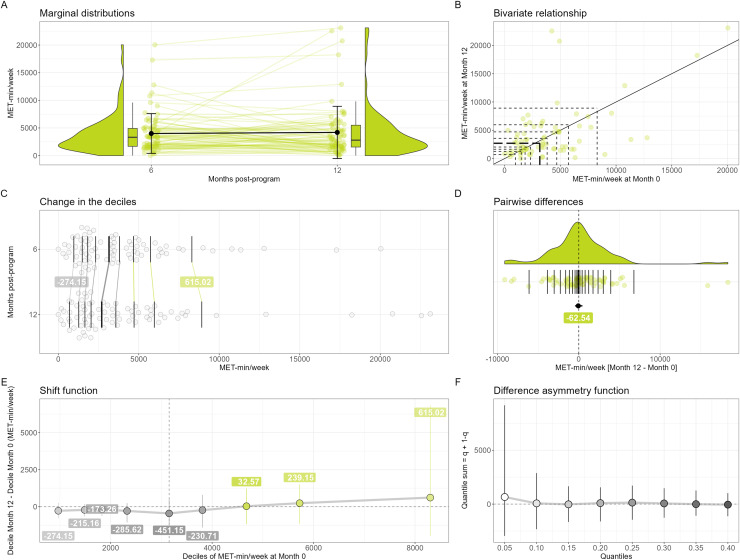
Change in IPAQ-SF MET-min/week between 6 and 12 months (*N* = 77). On (A), the errors bars over the points are the standard deviations around the means, while on (D) it is the percentile bootstrap 95% confidence interval around the median estimate. On (E) and (F), the error bars are percentile bootstrap 95% confidence intervals not corrected for multiple comparisons. On (B), (C) and (D), the horizontal and/or vertical segments are the estimates of the deciles (B and C) or the quantiles (D, step of 0.05) of the distributions; the thickest segments are the median estimates. On (B), the diagonal black line depicts the identity line. If any, significant results (based on adjusted *P* values) in the shift (E) and difference asymmetry (F) functions are highlighted using thick red circles. A small pseudo-random movement has been added horizontally and vertically to the raw data displayed on (B) to minimize the presence of points fully overlapped. The estimates of the deciles of the marginal distributions (B, C and D), the quantiles of the individual differences (D), the decile differences (E) and the quantile sums (F) have been computed using the Harrell-Davis estimator.

All 6MWT distances, IPAQ-SF MET-min/week and EMAPS scores measured during the study, and related descriptive statistics, are shown in figures and tables in Supplemental Material 3. Data and results regarding the change in 6MWT distance (0–12 months), IPAQ-SF MET-min/week (6–12 months) and EMAPS scores (0–12 months) are respectively shown in Figs. 1 and 2, and Supplemental Material 4. Results regarding the change in IPAQ-SF MET-min/week between 0 and 12 months are shown in Supplemental Material 5. Statistical details related to the shift and difference asymmetry functions are provided in Supplemental Material 6. Finally, details related to analyses of the participants with missing data at follow-up are provided in Supplemental Material 7, showing that no specific pattern was detected in their physical characteristics, exercise capacity, physical activity and motivation for physical activity compared to the remaining part of the initial sample of participants.

Regarding 6MWT distance, the 75 participants with complete data at both 0 and 12 months had a mean ± SD 6MWT distance of 605.44 ± 71.96 m at the end of the CR program. When analysing the change in 6MWT distance at 12 months post-program at the group level using the mean, we found a significant and trivial improvement compared to the end of the program (+12.56 m; *r* = 0.88; *t*(74) = 2.8614; *d*_*av*_ = 0.16; *P* = 0.005; Fig. 1A). However, our exploratory analyses failed to demonstrate that the change in the 6MWT distance distribution was uniform, with a significantly positive shift only for the 5^th^ (+17.42 m, *P* < 0.001), 6^th^ (+19.53 m, *P* < 0.001) and 7^th^ deciles (+19.61 m, *P* < 0.001) as shown in Fig. 1E. The typical individual change as assessed using the median [95% CI] of the differences (Fig. 1D) was also significantly positive (+13.25 [1.54 to 25.08] m), while the difference asymmetry function (Fig. 1F), with most of the quantile sums being significantly positive (*P* < 0.001) except the first one, revealed that the positive individual changes were larger than the negative ones except when comparing the most extreme quantiles. In other words, this series of results show that, at 12 months post-CR program, clinicians could expect having a slightly fitter group of patients when considering the average walking performance, but not when considering the lowest and highest fractions of the performances of the group. They also could expect a slightly better walking performance in a randomly chosen patient, as well as positive changes in the group that would be of greater magnitude than the negative changes.

Regarding IPAQ-SF scores, the 77 participants with complete IPAQ-SF data at both 6 and 12 months had a median (25^th^–75^th^) IPAQ-SF score of 3,318 (1,680–4,914) MET-min/week at 6 months after the end of the program. Our exploratory analyses did not reveal any change in physical activity level at the group level, with no statistically significant decile shift (Fig. 2E). Similarly, the typical individual change as assessed using the median [95% CI] of the differences (Fig. 2D) was not statistically significant (−62.54 [−457.28 to 424.54] MET-min/week), and the difference asymmetry function (Fig. 2F), showing no statistically significant quantile sums, did not reveal any imbalance between the magnitude of the negative and the positive individual changes at any level of the distribution of the individual changes. When analysing the change between 0 and 12 months (Supplemental Material 5), we could observe a significant decrease in MET-min/week when comparing the first seven deciles of the distributions and a typical negative change (−1,824.08 [−2,460.44 to 1,251.77] MET-min/week). For clinicians, this series of results means that when using the IPAQ-SF (MET-min/week), we have no evidence to expect any change in the group as a whole and in a randomly chosen patient bewteen 6 and 12 months post-CR program. However, clinicians could expect a group of patients or a randomly chosen patient to perform less physical activity at 12 months compared to when completing the last week of the CR program.

Concerning EMAPS scores, the 76 participants with complete EMAPS results at both 0 and 12 months post-CR program had a median (25^th^–75^th^) of 5.67 (5.00–6.00) for intrinsic motivation, 5.33 (4.00–6.00) for integrated regulation, 6.00 (5.67–6.67) for identified regulation, 4.17 (3.58–5.33) for introjected regulation, 1.00 (1.00–1.67) for external regulation and 1.00 (1.00–1.33) for amotivation at the end of the CR program. When considering the change in EMAPS scores at the group level, our exploratory analyses globally showed beneficial but possibly non-uniform changes in the different forms of motivation for physical activity, with significant decile shifts that were generally inferior to one point and only regarding the 5^th^ (+0.31, *P* = 0.045) and 6^th^ (+0.28, *P* < 0.001) deciles for intrinsic motivation, the 6^th^ (+0.55, *P* < 0.001) and 7^th^ (+0.49, *P* = 0.045) deciles for integrated regulation, the 5^th^ (+0.89), 6^th^ (+0.87), 7^th^ (+0.61) and 8^th^ (+0.37) deciles (*P* < 0.001) for introjected regulation, the 5^th^ (−0.04, *P* = 0.030) and 6^th^ (−0.31, *P* < 0.001) deciles for external regulation, and the 6^th^ (−0.02), 7^th^ (−0.24), 8^th^ (−0.85) and 9^th^ (−1.39) deciles (*P* < 0.001) for amotivation. However, when looking at the typical individual change as assessed using the median [95% CI] of the individual changes, it was not significant for intrinsic motivation (0.04 [−0.1 to 0.29]), for integrated regulation (+0.31 [−0.01 to 0.67]), for identified regulation (+0.11 [−0.08 to 0.32]), for external regulation (0 [−0.08 to 0]) and for amotivation (0 [0 to 0]). It was significant only for introjected regulation (+0.29 [0.01–0.69]) while remaining of little practical interest (less than 0.5 point on the seven-point EMAPS scale). Only quantile sums from the difference asymmetry function related to amotivation were significant, revealing a marked left-skewed distribution of the individual changes, with negative changes being larger than the positive ones, depicting a trend towards less amotivation. In summary, this series of results implies that clinicians could expect, after 12 months post-CR program, a beneficial change in the different forms of motivation when looking at the bulks of the distributions of patient scores but not when looking at the worst and best fractions of the distributions. It also means that they could expect a positive change in a randomly chosen patient only regarding introjected regulation and that the negative changes (decreases) would actually be larger than the positive changes (increases) in a group of patients only for amotivation.

**Figure 3:**
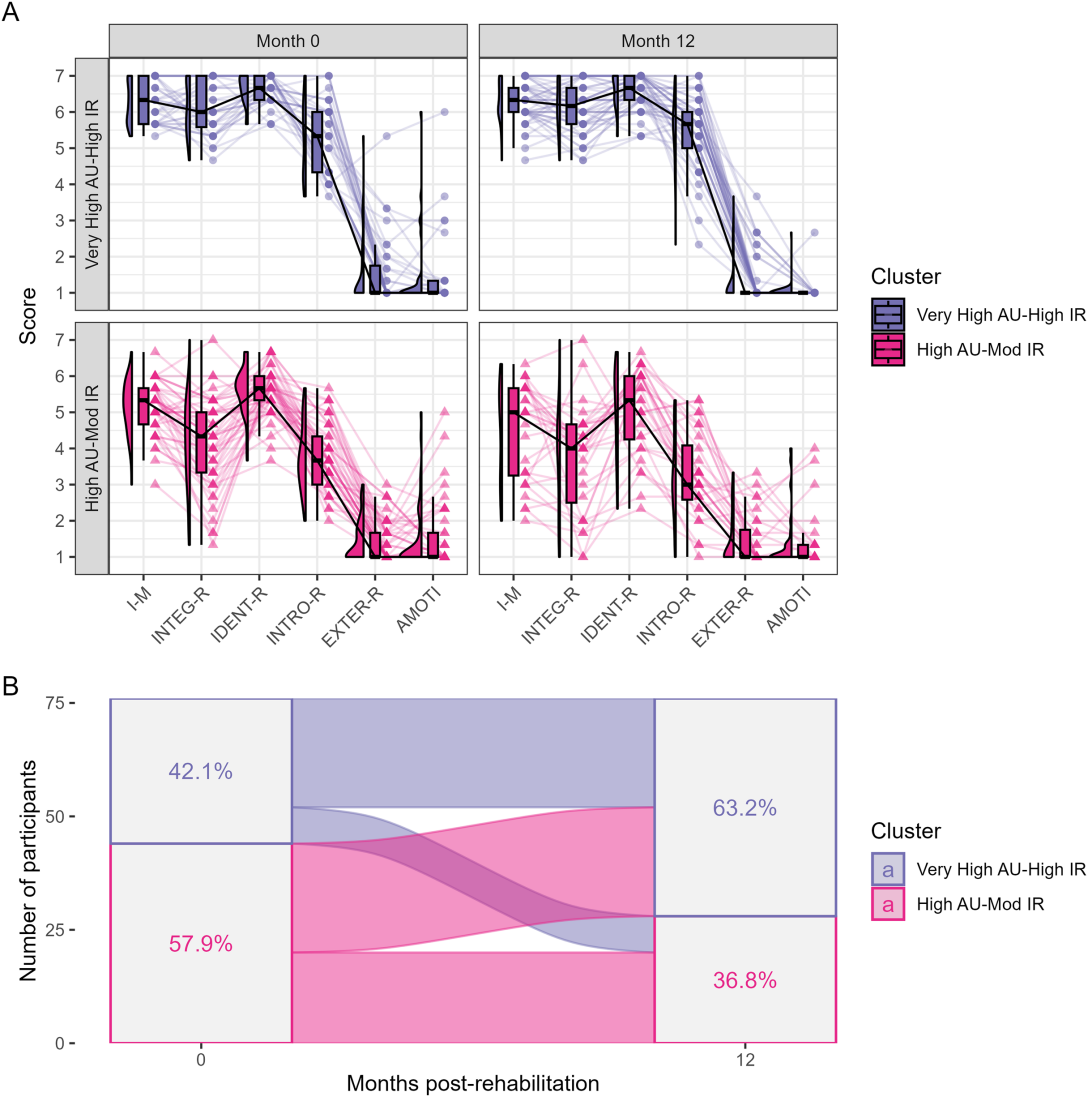
Change in physical activity motivational profile between the end of the CR program and 12 months after the program. (A) Shows the distributions of the EMAPS scores for each of the two identified motivational profiles at the end of the CR program (Month 0) and at the end of the follow-up period (Month 12). The multivariate distributions related to the two motivational profiles were overall significantly different both at the end of the program and at 12 months post-program, with significant differences for intrinsic motivation and all the forms of regulations excepted external regulation. (B) Shows the transition from a motivational profile to the other one between the end of the program and the end of the 12-month follow-up period. AU = autonomous motivation; I-M = intrinsic motivation; INTEG-R = integrated regulation; IDENT-R = identified regulation; INTRO-R/IR = introjected regulation; EXTER-R = external regulation; AMOTI = amotivation.

We identified two motivational profiles both at 0 and 12 months after the CR program among the 76 participants with complete EMAPS scores data (Fig. 3): a profile with a very high level of autonomous motivation and a high level of introjected regulation, thereafter named the ‘Very High Autonomous motivation (AU)-High Identified regulation (IR)’ profile; and a profile with a high level of autonomous motivation and a moderate level of introjected regulation, thereafter named the ‘High AU-Mod IR’ profile. Multivariate analysis suggested that the sets of the EMAPS scores from these two profiles were indeed different both at the end of the program and at 12 months post-program (*P* < 0.001), with significant differences for intrinsic motivation, integrated, identified and introjected regulations (*P* ≤ 0.05). The ‘High AU-Mod IR’ profile was more prevalent (57.9% of the participants) than the ‘Very High AU-High IR’ profile at the end of the program and inversely at 12 months post-CR program (36.8% *vs*. 63.2%). The proportions [95% CI] of participants related to a given scenario of cluster change were the followings: 57.9% [46.0–69.1%] of participants were associated to the same profile; 10.5% [4.7–19.7%] transited from the ‘Very High AU-High IR’ profile to the ‘High AU-Mod IR’ profile; and 31.6% [21.4–43.3%] transited from the ‘High AU-Mod IR’ profile to the ‘Very High AU-High IR’ profile (Fig. 3B). Importantly, information available in Supplemental Material 8 suggest the good consistency between the actual individual changes in motivational profile and the data-driven categories of profile change assigned to the study participants.

**Figure 4:**
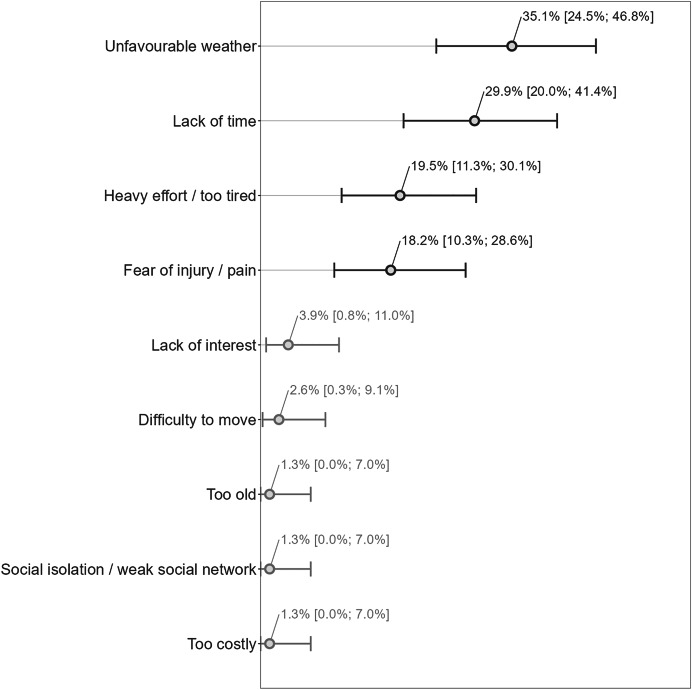
Barriers to physical activity (*N* = 77). Answers have been translated from French to English for the reader’s understanding of the figure. The main barriers have been highlighted with darker colours. Percentage estimates are provided with their respective 95% Clopper and Pearson confidence intervals.

The barriers to physical activity were mainly “unfavourable weather”, “lack of time”, “heavy effort/too tired”, and “fear of injury/pain” (in order of importance), with corresponding percentages of concerned participants being between 18% and 35% of the participants (*N* = 77), and the remaining items reaching proportions below 4% (Fig. 4).

**Figure 5:**
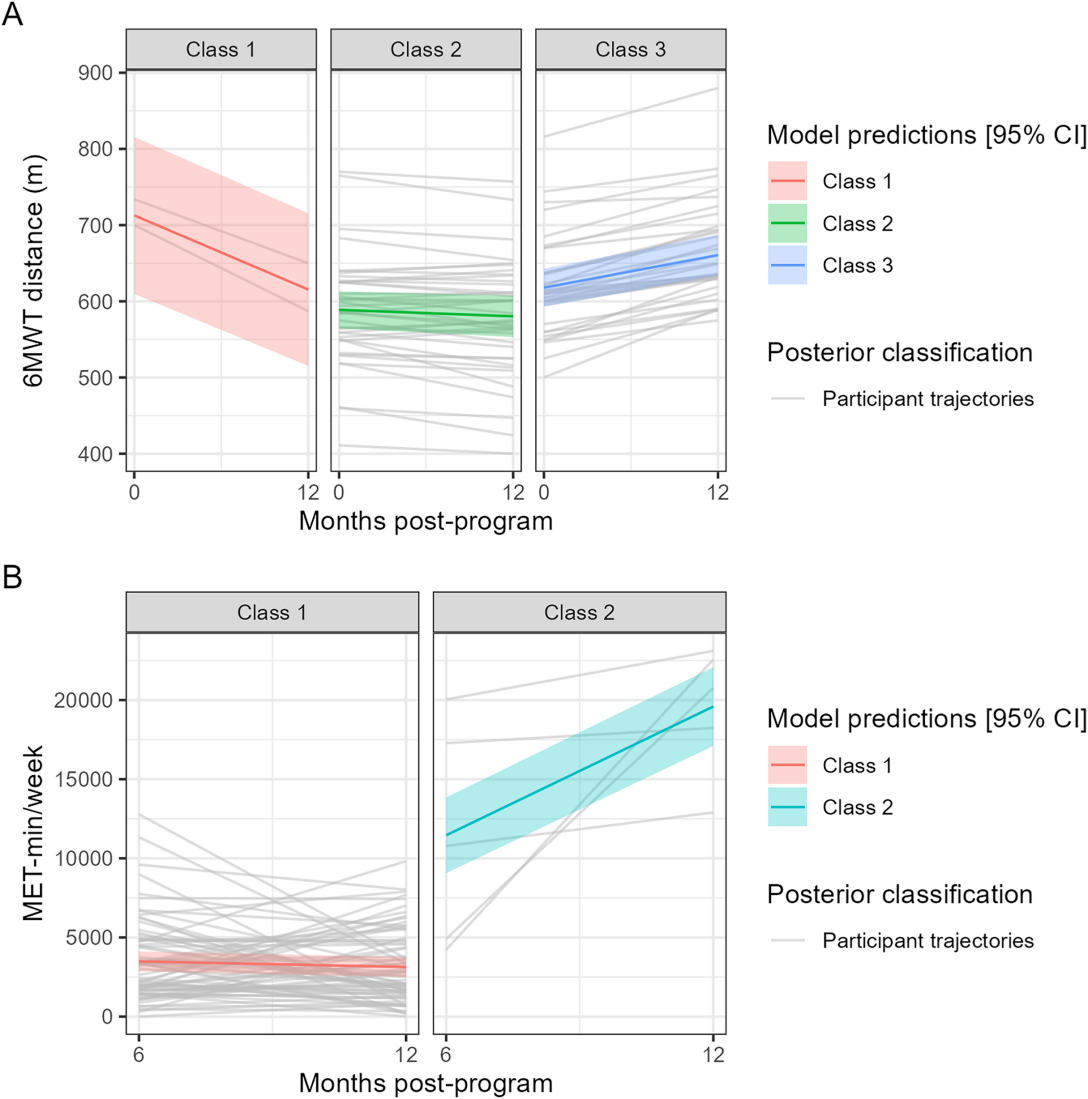
Predictions of the selected latent class linear mixed (trajectory) models along with the individual trajectories of the participants assigned to a given class of change. The models respectively for (A) 6MWT distance (0–12 months) and (B) IPAQ-SF MET-min/week (6–12 months).

Based on the latent class linear mixed model analysis (Fig. 5 and Supplemental Material 9), we considered that the change in 6MWT distance could be categorized into three classes (Fig. 5A) with the following class-specific effects of time (month) on 6MWT distance and related percentages of posterior classification of the participants: Class 1, significant decrease (−8.1 m/month, *P* < 0.001, *N* = 2 [2.7%]); Class 2, non-significant change (−0.7 m/month, *P* = 0.118, *N* = 39 [52.0%]); Class 3, significant increase (+3.6 m/month, *P* < 0.001, *N* = 34 [45.33%]). As shown in Fig. 5A, the model seemed to be able to globally propose correct classifications of the study participants. Regarding the analysis of IPAQ-SF MET-min/week (Fig. 5B), it was possible to get a categorization of the changes into two classes: Class 1, no significant change (−58.7 MET-min/week/month, *P* = 0.381, *N* = 72 [93.5%]); Class 2, significant increase (+1,355.8 MET-min/week/month, *P* < 0.001, *N* = 5, [6.5%]). Contrary to the model for 6MWT distance, the model for IPAQ-SF MET-min/week seemed to have a poorer performance of classification of the study participants, as shown in Fig. 5B (poor correspondence between the predictions of the model and the participant trajectories). None of the tested variables significantly predicted the assignment to a given class of change both for 6MWT distance and for IPAQ-SF MET-min/week.

## Discussion

The overall objective of the present study was to describe how exercise capacity, physical activity level and motivation for physical activity evolve over a long period of time (12 months) after a CR program in CHD patients, with a main focus on exercise capacity assessed using the 6MWT. Contrary to the previous studies, we were not interested in the change of scores over time compared to the results obtained at the entry in the CR program. The main results can be summarised as follows: there was a trivial and possibly non-uniform improvement in exercise capacity for the CHD patients at 12 months, both when considering the group as a whole and the individual trajectories throughout the follow-up period; we did not observe any significant change for physical activity level between 6 months and 12 months post-CR program both when considering the group as a whole and the individual trajectories, but a decrease in physical activity level could be observed at 12 months when compared with the end of the CR program; the changes in the different forms of motivation for physical activity were non-uniformly beneficial at the group level but without evidence of improvement when looking at the typical individual change for most of the motivational constructs; participants presented a motivational profile with high to very high levels of autonomous motivation and moderate to high levels of introjected regulation both at the end of the program and at 12 months post-program; the change in 6MWT distance and IPAQ-SF MET-min/week could be respectively categorized into three (decrease, no change, increase) and two (no change, increase) classes without the possibility to identify any predictor of the classification into a given class of change.

Previous studies compared follow-up measurements with the end of the CR program using either ≥1-yr post-program measurements with a laboratory maximal exercise test (Boesch et al., 2005; Nilsson et al., 2018) or ≤6-month post-program measurements with a 6MWT or a laboratory maximal exercise test (Giallauria et al., 2006; Pavy, Tisseau & Caillon, 2011; Ramadi et al., 2016). Regarding long-term follow-up studies, Nilsson et al. (2018) observed a statistically significant increase in mean peak oxygen uptake on the treadmill (+0.9 mL/min/kg; +2.5%) for the whole sample 1 year after the CR program, while Boesch et al. (2005) found a non-statistically significant change in mean maximal workload (+8.4 W; +5%) on the cycle ergometer 2 years after the CR program. Regarding the other studies, Ramadi et al. (2016) observed, 3 months after a “traditional” and a “fast track” program, respectively non-statistically significant mean changes of −5 m (−0.8%) and +15 m (+3%) in 6MWT distance. Pavy et al. (2011) did not observe at 6 months after the CR program a statistically significant change in 6MWT distance (mean change = 2 m; +0.3%). Finally, Giallauria et al. (2006) found, in patients discharged with general instructions, a statistically significant decrease in mean peak oxygen uptake on the cycle ergometer 3 months after the CR program (−2.3 mL/min/kg). Overall, while previous studies showed mixed results regarding statistically significant changes in exercise capacity over time after a CR program, the magnitude of the variations appeared relatively small, both a few months and 1 to 2 years after the CR program, as in the present study. Beyond the fact the mean change in 6MWT distance we found at 12 months seems to be relatively small (12.56/605.44 = 2%), it could be difficult to consider that change as clinically relevant for the group of patients since it likely remained within the learning effect zone of the 6MWT, that is a test-retest mean change between 2% and 8% with tests separated by several days (the between-test duration actually is unclear in the literature) (Bellet, Adams & Morris, 2012). That being said, it is unknown whether a learning effect is actually maintained 1 year after a previous measurement in CHD patients.

The small positive change we found in 6MWT distance actually could be considered as an encouraging result regarding the interest of the CR program to allow a given group of CHD patients to maintain the highest walking capacity as possible over time, in particular when considering the possible trajectory of 6MWT distance observed in other studies, including those with other clinical populations or older adults. Indeed, Oerkild et al. (2011) observed in cardiac patients (≥65 years), who had followed a program in centers and at home, a decrease respectively of −27 m (baseline: 376 m) and −45 m (baseline: 346 m) in mean 6MWT distance between 3 and 12 months after the end of the program. In older adults, Manning et al. (2024) observed at 12 months a mean increase in 6MWT distance of about 40 m in active older adults (*N* = 318; 72.5 yr on average; exercise 3 days/week; baseline 6MWT distance: 457 m) and a mean decrease of about 76 m in sedentary older adults (*N* = 146; 74.4 yr on average, no moderate exercise in the past 5 yrs; baseline 6MWT distance: 421 m). Finally, in chronic obstructive pulmonary disease (COPD) patients, Frei et al. (2022) observed at 12 months, in the control group (*N* = 51; routine care; baseline 6MWT distance: 417 m) of a randomized trial, a mean decrease of −24 m in 6MWT distance. While the results of the CR program we implemented compare well with several studies that reported changes in 6MWT distance at 1 yr, especially considering the relatively high 6MWT distances of our participants who were *de facto* more prone to a decrease at 1 yr, it seems possible for a cohort of patients to have a maintained 6MWT distance at 1 yr without having followed a rehabilitation program, as previously observed in COPD patients (*N* = 294; baseline: 388 m; median age: 66 yr) (Casanova et al., 2007).

Interestingly, beyond the positive mean change in 6MWT distance, our results suggest an incomplete shift of the whole distribution of patient results towards better walking performances. Indeed, while the top part of the bulk of the distribution (5th, 6th, and 7th deciles) had a significant positive shift of similar magnitude for the different deciles, the other decile shifts were not significant. In particular, the first decile of the distribution at 12 months had a non-statistically significant negative shift compared to the first decile at the end of the program, meaning the lowest walking performances at 12 months were lower than at the end of the program. This result could invite to conduct confirmatory studies to more precisely investigate the possible non-uniformity of the change in exercise capacity.

Regarding physical activity, while the individual changes between 6 and 12 months could be substantial (approximately ranging from −10,000 to >15,000 MET-min/week), we did not find any statistically significant change in the deciles nor in the typical individual change of the MET-min/week. This result is in line with Ramadi et al. (2016) who did not find a change in any physical activity parameters measured using the SenseWear Armband multi-sensor device but after 3 months post-program only.

The EMAPS scores we have obtained were in line with previous results obtained in persons recruited in health centers providing physical activity rehabilitation, including people of various ages or having a chronic condition (Boiché et al., 2019). When analysed throughout a clustering approach, these scores depicted motivational profiles with high to very high levels of autonomous forms of motivation in combination with moderate to high levels of introjected regulation both at the end of the CR program and 12 months after the program. These profiles compare very well with the most self-determined motivational profiles that have been previously identified in the work domain (Gillet, Berjot & Paty, 2010), the sport domain (Gillet, Vallerand & Rosnet, 2009) and the public health domain (Friederichs et al., 2015). Several procedures were implemented during our CR program so that patients develop autonomous forms of motivation for physical activity during the program. In particular, the number of lifestyle counselling sessions, as well as the discovery of various physical activities during the program so that patients may find enjoyable activities that could fit their preferences, might have played a role in the relatively good scores the patients obtained. Moreover, since a focus was made on future lifestyle and not particularly on exercise adherence during the CR program, it could be expected that autonomous motivation for physical activity at the end of the program would be at least maintained over time.

To our knowledge, one study investigated the evolution of motivation for physical activity over time in cardiac patients after a CR program (Kim et al., 2021b). Contrary to the overall positive shift of the motivation scores found in the present study, Kim et al. (2021b) have found a decrease in motivation for physical activity at 9 months post-program as assessed using the Physical Activity and Leisure Motivation Scale. Thus, from this perspective, the general positive shift as well as the close-to-zero medians of the individual changes for the different motivation variables, combined with the fact that the participants stayed in a motivational profile that was highly to very highly self-determined, could be considered as encouraging outcomes of the CR program implemented in the present study since autonomous motivation for physical activity is a powerful driver of long-term exercise behaviour in CHD patients (Slovinec D’Angelo et al., 2014). Further studies and analyses should be conducted to confirm these results and to characterize the change in motivation for physical activity over time in more heterogeneous physical activity motivational profiles that could be identified at the end of a CR program.

At the end of the follow-up period (12 months post-program), the main barriers to physical activity were an unfavourable weather, a lack of time, the fact to be too tired, and the fear of injury. The lack of time appears to be an important and consistent barrier to physical activity when comparing with similar works (Brown, 1999; Fleury et al., 2004). Moreover, the relatively high proportions of participants acknowledging intrapersonal barriers to physical activity (*i.e*., lack of time, the fact to be too tired, and the fear of injury) compared to interpersonal barriers (*i.e*., social isolation/weak social network) or to organizational barriers (*i.e*., financial costs) are in line with the results from the Fleury et al. study (2004) who found, using a qualitative approach (an open-ended question) and a categorization of the answers based on the social ecological framework, that intrapersonal factors were the most reported barriers to physical activity maintenance in CHD patients.

We took the opportunity of having both functional (6MWT distance), behavioural (IPAQ-SF MET-min/week) and motivational (EMAPS-based clusters and barriers to physical activity) data to explore, *via* a latent class trajectory modelling analysis, the possibility to determine the predictors of particular classes of changes both in exercise capacity (6MWT distance) and physical activity (IPAQ-SF MET-min/week) over time. This analysis could add a value compared to the decile shift analysis we conducted by more clearly highlighting the different profiles of changes in the outcomes of interest, thus reinforcing the idea that patients may have different needs to get the best trajectory as possible for a given outcome after a CR program. A few previous studies had already identified different clusters of trajectories in physical activity outcomes over time after a CR program (Sweet et al., 2011; Blanchard et al., 2014; Limpens et al., 2023) but to our knowledge not for exercise capacity. Based on these studies, it could be expected that the baseline motivational profile (Sweet et al., 2011), exercise capacity and physical activity (Blanchard et al., 2014; Limpens et al., 2023) here measured at the end of the CR program in the present study, are identified as predictors at least of physical activity (here between 6 and 12 months post-CR program). Unfortunately, we were not able to identify any predictor that could allow clinicians to anticipate the trajectory a patient would be likely to have after a CR program depending on their profile. The relatively high level of 6MWT distance for all participants, the potentially high measurement error related to the IPAQ-SF and the relative lack of differences between the motivational profiles of our participants were clearly not in favour of their identification as potential predictors of a particular change in exercise capacity or physical activity after the CR program. Of note, our relatively low sample size to conduct such exploratory analyses imposed us important constraints in the definition of the structure of the models (*e.g*., impossibility to get models converging with several covariates when using random effects on slopes), which is line with the fact that such a kind of analyses generally require several hundreds of participants to ensure robust results (Sinha, Calfee & Delucchi, 2021). For these reasons, the results from these analyses should be considered with great caution.

As a general note, the possible influence of the relatively high physical activity dose experienced by the participants during the CR program on the outcomes of the present study should be considered in the comparisons with past and future studies. Our CR program included approximately up to 15 sessions of physical activity per week, including the structured endurance exercise sessions (4 per week) and resistance exercise sessions (2–3 per week) recommended by the French Society of Cardiology (Pavy et al., 2011). Such a dose of physical activity could have had a particular influence on future physical activity behaviour compared to programs with less physical activity sessions since a given amount of structured exercises in a CR program leads to an increase in self-efficacy for various physical activities (Cheng & Boey, 2002) and self-efficacy mediates the relationship between motivation and physical activity behaviour (Klompstra, Jaarsma & Strömberg, 2018).

The present study has several strengths. First, it adds new data to a very scarce literature related to the long-term change in exercise capacity and physical activity, but also motivation for physical activity, after a CR program in CHD patients. Second, the present study introduces for the first time an analysis considering not only the change in the central tendency, but also the change in the quantiles of the distribution of the considered outcome coupled with an analysis of the individual changes. This is important because clinicians are likely to be interested in the success of an intervention for a whole cohort of patients or a random patient from a given cohort, not only for an average patient. Such an approach allowed in the present study to observe non-uniform shifts of the different parts of the outcome distributions, with typical individual changes that did not always reflect the positive changes observed at the group level, in particular regarding motivation for physical activity. These observations need, however, to be confirmed with appropriately powered studies. Third, all the materials have been made available in a GitHub repository with detailed explanations about how to reproduce the analytical pipeline of the project, letting the opportunity to verify all the analyses and to compute additional statistics if needed.

The present study also has several limits. First, we used the 6MWT to depict exercise capacity. As the 6MWT remains a submaximal exercise task that is moderately correlated with maximal oxygen uptake, our results may not be valid to describe the change in some maximal physiological parameters and thus should be rather used to reflect on walking capacities. Second, the 6MWT performances were on average large at the end of the CR program compared to previous works (Pavy, Tisseau & Caillon, 2011; Ramadi et al., 2016; Racodon, Porrovecchio & Pezé, 2019), suggesting the present sample of patients may show levels of performance that are not really representative of a typical CHD patient. Moreover, several factors known to influence physical behaviour and motivation for physical activity, as employment status or years of education (Fleury et al., 2004), were not measured in the present study, which hides the precise characteristics of the population that is actually concerned by the present results. Third, as we used an observational design with one group only, it is not possible to associate the observed changes only to the pure impact of the CR program and not to other unknown factors. Fourth, we used a French version of the IPAQ-SF with unclear psychometric properties, and some MET-min/week scores appeared surprisingly high for people with a chronic disease, thus questioning the validity of the measurements. Even if such high scores should have a minor impact on our results since they were based on quantile analyses, for these reasons the IPAQ-SF results should be used with caution, and future studies should prefer device-based methods to more accurately investigate the shift in physical activity level in CHD patients. Moreover, we could not use the comparisons of the measurements performed at 12 months *vs*. the end of the CR program to describe the change in natural physical activity behaviour since the first measurement with the IPAQ-SF was not related to a week after patient discharge. Future studies should consider a measurement during the days following the end of the CR program to capture the patient behaviour in their natural environment and then to make meaningful analyses of behaviour change over time. Fifth, while several exploratory analyses led to several statistically significant results, in particular several changes in the deciles of several variables of interest, a lot of other decile changes were not statistically significant, likely because of a lack of statistical power to analyse such a number of changes while controlling for the Type I error rate. Sixth, precise comparisons with similar works conducted in CHD patients are difficult since we used different tools to assess the outcomes of interest, in particular physical activity, motivation for physical activity and barriers to physical activity. Seventh, the lack of information regarding participants’ medications during the follow-up period prevents a full understanding of the context in which the results were obtained. Eight, 12 months follow-up could be considered as a not so long follow-up duration and may not accurately depict the maintenance or the change of patient characteristics over several years. Finally, the weak representation of women in the present study makes the generalization of the findings to women difficult. This is largely related to the lack of women who attended the CR program. Such a lack of attendance has previously been highlighted in the scientific literature and could be related to the reported difficulties from women to meet social costs of participation to CR programs, including dealing with domestic and family demands, while women would report little support from their communities and significant others (Clark et al., 2012). In France, this underrepresentation of women could also be partly related to their large underrepresentation in people hospitalized following a CHD-related event (Fourcade et al., 2017). Future researches on the present topic should consider strategies to allow a higher representation of women in clinical trials and thus to contribute to a better health equity (Van Diemen et al., 2021).

## Conclusions

The present study investigated the change in exercise capacity (6MWT distance), physical activity level (IPAQ-SF MET-min/week) and motivation for physical activity (EMAPS scores) using approaches that considered both the group as a whole and the individual trajectories between the time points of interest. Both the approaches revealed a trivial and possibly non-uniform improvement in exercise capacity of CHD patients 12 months after a CR program, but failed to highlight any change for the whole group and a typical individual regarding physical activity level between 6 months and 12 months post-program. The conclusions related to motivation for physical activity are mixed, with possibly non-uniform changes at the group level towards higher degrees of the most autonomous forms of motivation (intrinsic motivation, integrated regulation, identified regulation) and introjected regulation, and towards lower degrees of external regulation and amotivation, but with no evidence of a typical individual change for most of the motivational constructs. These results regarding the different forms of motivation translated into motivational profiles that could be described as presenting high to very high levels of autonomous motivation and moderate to high levels of introjected regulation in the participants both at the end of the program and at 12 months after the program. Moreover, we observed that at 12 months post-program, CHD patients mainly reported barriers to physical activity related to unfavourable weather, lack of time, the fact to be too tired, and fear of injury. While different classes of changes in exercise capacity and physical activity could be identified, none of the tested variables (end-program exercise capacity, end-program physical activity level, end-program motivational profile, unfavourable wether and lack of time as main barriers to physical activity) predicted the assignment to a given class of change. Further work is needed to more precisely analyse the shift in the quantiles of 6MWT, IPAQ-SF and EMAPS scores as well as the predictors of the classes of changes in exercise capacity and physical activity in a sample of patients who could be more representative of the population of patients with CHD who follow a CR program.

## Supplemental Information

10.7717/peerj.18885/supp-1 Supplemental Information 1 French version of the IPAQ-SF provided in the Pralimap trial.

10.7717/peerj.18885/supp-2 Supplemental Information 2 Analysis performed to compute the required sample size.

10.7717/peerj.18885/supp-3 Supplemental Information 3 Data related to all the 6MWT, IPAQ-SF, and EMAPS measurements during the study.

10.7717/peerj.18885/supp-4 Supplemental Information 4 Figures related to the change in EMAPS scores between 0 and 12 months (N = 76).

10.7717/peerj.18885/supp-5 Supplemental Information 5 Change in IPAQ-SF MET-min/week between 0 and 12 months.

10.7717/peerj.18885/supp-6 Supplemental Information 6 Data related to the shift and difference asymmetry functions to describe the change in 6MWT distance (0-12 months), IPAQ-SF MET-min/week (6-12 months; 0-12 months), and EMAPS scores (0-12 months).

10.7717/peerj.18885/supp-7 Supplemental Information 7 Characteristics of the participants with missing data at 12 months.

10.7717/peerj.18885/supp-8 Supplemental Information 8 The changes in the EMAPS scores associated to the different scnearii of change in the motivational profile between 0 and 12 months after the CR program.

10.7717/peerj.18885/supp-9 Supplemental Information 9 Models to analyse the classes of changes in 6MWT distance and IPAQ-SF MET-min/week and their potential predictors (note: the configuration of the 3-latent class mixed model shown for 6MWT is the one before the permutation of the order of the classes with.

10.7717/peerj.18885/supp-10 Supplemental Information 10 Results shown in the article.

10.7717/peerj.18885/supp-11 Supplemental Information 11 STROBE checklist.

## Additional Information and Declarations

### Data Availability

The following information was supplied regarding data availability:

All the instructions and materials allowing the reproduction of the analyses and the results are available at GitHub and Open Science Framework:

- https://github.com/pydemull/APACo

- de Müllenheim, Pierre-Yves. 2024. “APA&Co.” OSF. doi:10.17605/OSF.IO/S2XWU.

The Supplemental Files are also available at Open Science Framework: de Müllenheim, Pierre-Yves. 2024. “Supplemental Materials.” OSF. doi:10.17605/OSF.IO/FPCRV.

The results of the study were obtained using several R packages:

• Initial tasks of the data analysis workflow (import, tidy, and transform data): {tidyverse} (Wickham et al., 2019); {tidyselect} (Henry & Wickham, 2024a).

• Figures: {ggplot2} (Wickham, 2016), {Hmisc} (Harrell, 2024), {patchwork} (Lin Pedersen, 2024), {ggbeeswarm} (Clarke, Sherrill-Mix & Dawson, 2023), {ggrepel} (Slowikowski, 2023), {rlang} (Henry & Wickham, 2024b), {ragg} (Lin Pedersen & Shemanarev, 2024), {ggrain} (Allen et al., 2021), {ggalluvial}

• Interactive tables displayed in the Supplemental Materials: {reactable} (Lin, 2023).

• html format-based Supplemental Materials: {rmarkdown} (Xie, Grolemund & Allaire, 2023).

• Descriptive statistics: {skimr} (Waring et al., 2022); {questionr} (Barnier, Briatte & Larmarange, 2023).

• t-test, Pearson correlation coefficient, *P* value adjustement, and confidence intervals for proportions: {stats} (R Core Team, 2023).

• Decile differences analysis: {rogme} (Rousselet, Pernet & Wilcox, 2017).

• Variable scaling : {stats} (R Core Team, 2023).

• Multivariate outlier analysis: {performance} (Lüdecke et al., 2021; Thériault et al., 2024).

• Clustering analysis: {stats} (R Core Team, 2023), {factoextra} (Kassambara & Mundt, 2020), {cluster} (Maechler et al., 2023).

• Nonparametric multivariate analysis: {npmv} (Burchett et al., 2017).

• Latent class trajectory modelling: {lcmm} (Proust-Lima, Philipps & Liquet, 2017).

• Analytical pipeline implementation: {target} (Landau, 2021a), {tarchetypes} (Landau, 2021b).
