# Supplementary material for: Change in exercise capacity, physical activity and motivation for physical activity at 12 months after a cardiac rehabilitation program in coronary heart disease patients: a prospective, monocentric and observational study
Source: PeerJ. 2025 Feb 14;13:e18885. doi: 10.7717/peerj.18885 (PMC11831972; doi:10.7717/peerj.18885)
Supplement: Supplemental Information 1 [file peerj-13-18885-s001.pdf]

IPAQ

International Physical Activity Questionnaire

(Version française juillet 2003)

Nous nous intéressons aux différents types d'activités physiques que vous faites dans votre vie quotidienne. Les questions suivantes portent sur le temps que vous avez passé à être actif physiquement au cours des **7 derniers jours**. Répondez à chacune de ces questions même si vous ne vous considérez pas comme une personne active. Les questions concernent les activités physiques que vous faites au lycée, lorsque vous êtes chez vous, pour vos déplacements, et pendant votre temps libre.

Bloc 1 : Activités intenses des 7 derniers jours

1. Pensez à toutes les **activités intenses** que vous avez faites au cours des **7 derniers jours**.
Les activités physiques intenses font référence aux activités qui vous demandent un effort physique important et vous font respirer beaucoup plus difficilement que normalement. Pensez seulement aux activités que vous avez effectuées pendant **au moins 10 minutes d'affilée.**

1-a. Au cours des **7 derniers jours**, **combien y a-t-il eu de jours** au cours desquels vous avez fait des **activités physiques intenses** comme porter des charges lourdes, bêcher, faire du VTT ou jouer au football ?

|__|__| **jour(s)**

❒ Je n'ai pas eu d'activité physique intense ➲ ***Passez au bloc 2***

1-b. Au total, combien de **temps** avez-vous passé à faire des **activités intenses au cours des 7 derniers jours ?**

|__|__| **heure(s)** |__|__| **minutes**

❒ Je ne sais pas

Bloc 2 : Activités modérées des 7 derniers jours

2. Pensez à toutes les **activités modérées** que vous avez faites au cours des **7 derniers jours**.
Les activités physiques modérées font référence aux activités qui vous demandent un effort physique modéré et vous font respirer un peu plus difficilement que normalement. Pensez seulement aux activités que vous avez effectuées pendant **au moins 10 minutes d'affilée.**

2-a. Au cours des **7 derniers jours**, **combien y a-t-il eu de jours** au cours desquels vous avez fait des **activités physiques modérées** comme porter des charges légères, passer l'aspirateur, faire du vélo tranquillement ou jouer au volley-ball ? Ne pas inclure la marche.

|__|__| **jour(s)**

❒ Je n'ai pas eu d'activité physique modérée ➲ ***Passez au bloc 3***

2-b. Au total, combien de **temps** avez-vous passé à faire des **activités modérées au cours des 7 derniers jours ?**

|__|__| **heure(s)** |__|__| **minutes**

❒ Je ne sais pas

Bloc 3 : La marche des 7 derniers jours

3. Pensez au temps que vous avez passé à **marcher au moins 10 minutes d'affilée** au cours des **7 derniers jours**.
Cela comprend la marche au lycée et à la maison, la marche pour vous rendre d'un lieu à un autre, et tout autre type de marche que vous auriez pu faire pendant votre temps libre pour la détente, le sport ou les loisirs.

3-a. Au cours des **7 derniers jours**, **combien y a-t-il eu de jours** au cours desquels vous avez marché pendant **au moins 10 minutes d'affilée.**

|__|__| **jour(s)**

❒ Je n'ai pas fait de marche ➲ ***Passez au bloc 4***

3.b. Au total, combien d'épisodes de marche d'au **moins 10 minutes d'affilée**, avez-vous effectué au cours des **7 derniers jours** ?

|__|__|__|__| **nombre d'épisodes de 10 minutes d'affilée**

Exemples :

Lundi :	1 marche de 60 minutes		6 épisodes
Mardi :	1 marche de 20 minutes et 3 marches de 5 minutes		2 épisodes
Mercredi :	1 marche de 35 minutes		3 épisodes
Jeudi :	1 marche de 8 minutes		0 épisode
Vendredi :	1 marche de 6 minutes puis 3 marches de 4 minutes	→	0 épisode
Samedi :	1 marche de 18 minutes		1 épisode
Dimanche :	1 marche de 10 minutes et 3 marches de 5 minutes		1 épisode
		Total	13 épisodes

❒ Je ne sais pas

Bloc 4 : Temps passé assis au cours des 7 derniers jours

4. La dernière question porte sur **le temps que vous avez passé assis** pendant les jours de semaine, au cours des **7 derniers jours**. Cela comprend le temps passé assis au lycée, à la maison, lorsque vous étudiez et pendant votre temps libre. Il peut s'agir par exemple du temps passé assis à un bureau, chez des amis, à lire, à être assis ou allongé pour regarder la télévision, devant un écran.

4-a. Au cours des **7 derniers jours**, pendant les jours de semaine, **combien de temps**, en moyenne, avez vous passé **assis** ?

|__|__| **heure(s)** |__|__| **minutes**

❒ Je ne sais pas
