# Supplementary material for: Change in exercise capacity, physical activity and motivation for physical activity at 12 months after a cardiac rehabilitation program in coronary heart disease patients: a prospective, monocentric and observational study
Source: PeerJ. 2025 Feb 14;13:e18885. doi: 10.7717/peerj.18885 (PMC11831972; doi:10.7717/peerj.18885)
Supplement: Supplemental Information 2 [file peerj-13-18885-s002.docx]

**SM2. Analysis performed to compute the required sample size**


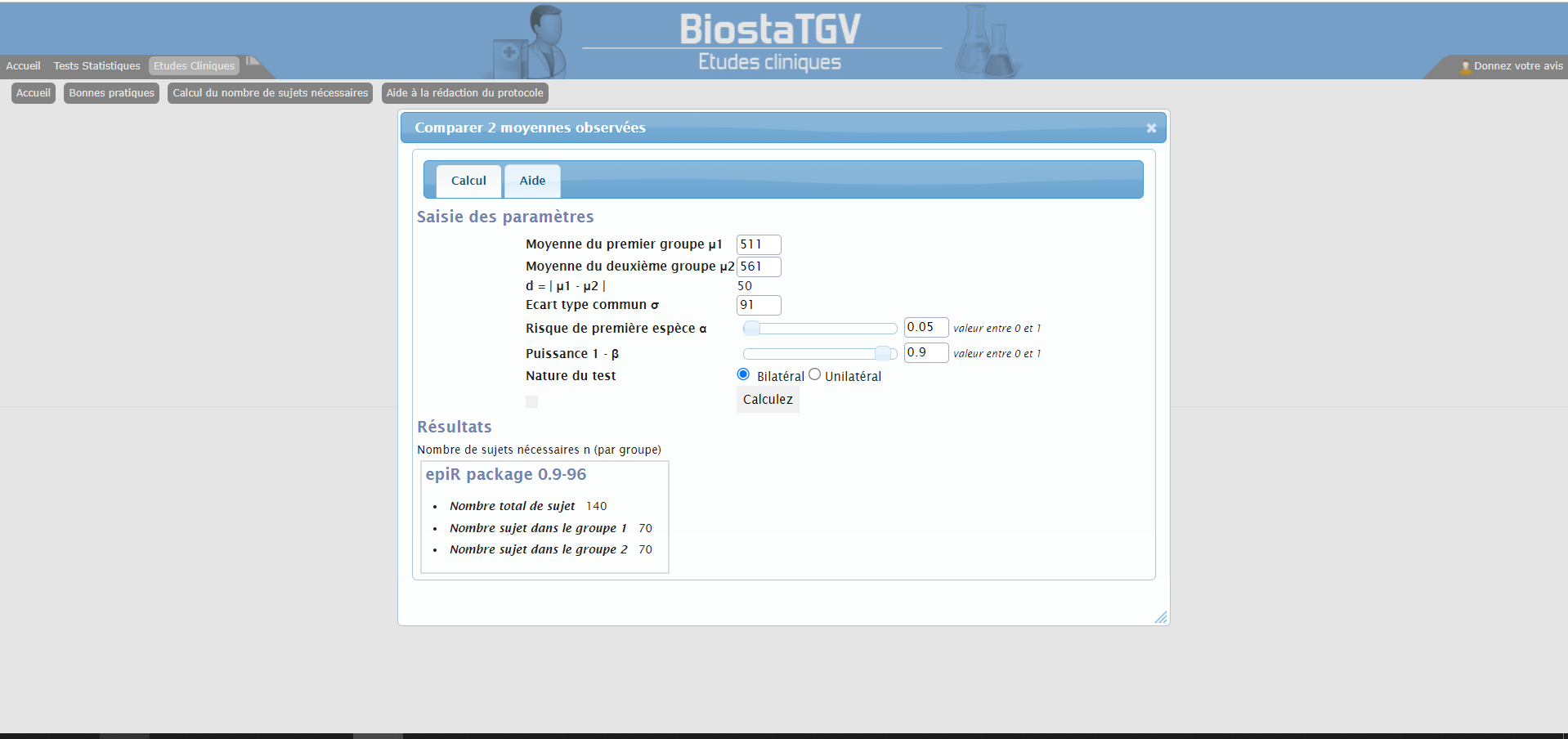


*Note.* The required sample size for the APA&Co study was obtained by dividing the result of the analysis (*N*=140) by 2. Please see the Methods section of the manuscript for the details of the explanations.
