# Supplementary material for: Change in exercise capacity, physical activity and motivation for physical activity at 12 months after a cardiac rehabilitation program in coronary heart disease patients: a prospective, monocentric and observational study
Source: PeerJ. 2025 Feb 14;13:e18885. doi: 10.7717/peerj.18885 (PMC11831972; doi:10.7717/peerj.18885)
Supplement: Supplemental Information 3 [file peerj-13-18885-s003.html]

APA&Co project | SM3. Data related to all the 6MWT, IPAQ-SF, and EMAPS measurements during the study


## Table of content

Code 

- Show All Code
- Hide All Code

# APA&Co project | SM3. Data related to all the 6MWT, IPAQ-SF, and EMAPS measurements during the study

# 1 Figures

## 1.1 6MWT

```
targets::tar_read(p_6MWT_all)
```

## 1.2 IPAQ-SF

```
targets::tar_read(p_IPAQ_all)
```

## 1.3 EMAPS

```
targets::tar_read(p_EMAPS_all)
```

# 2 Descriptive statistics

```
targets::tar_read(table_all_desc_stat)
```
