# Supplementary material for: Change in exercise capacity, physical activity and motivation for physical activity at 12 months after a cardiac rehabilitation program in coronary heart disease patients: a prospective, monocentric and observational study
Source: PeerJ. 2025 Feb 14;13:e18885. doi: 10.7717/peerj.18885 (PMC11831972; doi:10.7717/peerj.18885)
Supplement: Supplemental Information 4 [file peerj-13-18885-s004.html]

APA&Co project | SM4. Figures related to the change in EMAPS scores between 0 and 12 months (N = 76)


## Table of content

Code 

- Show All Code
- Hide All Code

# APA&Co project | SM4. Figures related to the change in EMAPS scores between 0 and 12 months (N = 76)

```
targets::tar_load(change_EMAPS)
res <-
  purrr::map(change_EMAPS, function(x) {
  
  knitr::knit_child(text = c(
                    
                    "\n",
                    "# `r x$variable`",
                    "\n",
                    "```{r, echo = FALSE, fig.width = 20, fig.height = 20, dpi = 250, fig.cap = 'On panel A, the errors bars over the points are the standard deviations around the means, while on panel D it is the percentile bootstrap 95% confidence interval around the median estimate. On panels E and F, the error bars are percentile bootstrap 95% confidence intervals not corrected for multiple comparisons. On panels B, C and D, the horizontal and/or vertical segments are the estimates of the deciles (panels B and C) or the quantiles (panel D, step of 0.05) of the distributions; the thickest segments are the median estimates. On panel B, the diagonal black line depicts the identity line. If any, significant results (based on adjusted *P* values) in the shift (panel E) and difference asymmetry (panel F) functions are highlighted using thick red circles. A small pseudo-random movement has been added horizontally and vertically to the raw data displayed on panel B to minimize the presence of points fully overlapped. The estimates of the deciles of the marginal distributions (panels B, C and D), the quantiles of the individual differences (panel D), the decile differences (panel E) and the quantile sums (panel F) have been computed using the Harrell-Davis estimator.'}",
                    "print(x$p)",
                    "```"
  ),
  envir = environment(),
  quiet  = TRUE
  )
})

cat(unlist(res), sep = "\n")
```

# 1 Intrinsic motivation score

On panel A, the errors bars over the points are the standard deviations
around the means, while on panel D it is the percentile bootstrap 95%
confidence interval around the median estimate. On panels E and F, the
error bars are percentile bootstrap 95% confidence intervals not
corrected for multiple comparisons. On panels B, C and D, the horizontal
and/or vertical segments are the estimates of the deciles (panels B and
C) or the quantiles (panel D, step of 0.05) of the distributions; the
thickest segments are the median estimates. On panel B, the diagonal
black line depicts the identity line. If any, significant results (based
on adjusted *P* values) in the shift (panel E) and difference
asymmetry (panel F) functions are highlighted using thick red circles. A
small pseudo-random movement has been added horizontally and vertically
to the raw data displayed on panel B to minimize the presence of points
fully overlapped. The estimates of the deciles of the marginal
distributions (panels B, C and D), the quantiles of the individual
differences (panel D), the decile differences (panel E) and the quantile
sums (panel F) have been computed using the Harrell-Davis estimator.

# 2 Integrated regulation score

On panel A, the errors bars over the points are the standard deviations
around the means, while on panel D it is the percentile bootstrap 95%
confidence interval around the median estimate. On panels E and F, the
error bars are percentile bootstrap 95% confidence intervals not
corrected for multiple comparisons. On panels B, C and D, the horizontal
and/or vertical segments are the estimates of the deciles (panels B and
C) or the quantiles (panel D, step of 0.05) of the distributions; the
thickest segments are the median estimates. On panel B, the diagonal
black line depicts the identity line. If any, significant results (based
on adjusted *P* values) in the shift (panel E) and difference
asymmetry (panel F) functions are highlighted using thick red circles. A
small pseudo-random movement has been added horizontally and vertically
to the raw data displayed on panel B to minimize the presence of points
fully overlapped. The estimates of the deciles of the marginal
distributions (panels B, C and D), the quantiles of the individual
differences (panel D), the decile differences (panel E) and the quantile
sums (panel F) have been computed using the Harrell-Davis estimator.

# 3 Identified regulation score

On panel A, the errors bars over the points are the standard deviations
around the means, while on panel D it is the percentile bootstrap 95%
confidence interval around the median estimate. On panels E and F, the
error bars are percentile bootstrap 95% confidence intervals not
corrected for multiple comparisons. On panels B, C and D, the horizontal
and/or vertical segments are the estimates of the deciles (panels B and
C) or the quantiles (panel D, step of 0.05) of the distributions; the
thickest segments are the median estimates. On panel B, the diagonal
black line depicts the identity line. If any, significant results (based
on adjusted *P* values) in the shift (panel E) and difference
asymmetry (panel F) functions are highlighted using thick red circles. A
small pseudo-random movement has been added horizontally and vertically
to the raw data displayed on panel B to minimize the presence of points
fully overlapped. The estimates of the deciles of the marginal
distributions (panels B, C and D), the quantiles of the individual
differences (panel D), the decile differences (panel E) and the quantile
sums (panel F) have been computed using the Harrell-Davis estimator.

# 4 Introjected regulation score

On panel A, the errors bars over the points are the standard deviations
around the means, while on panel D it is the percentile bootstrap 95%
confidence interval around the median estimate. On panels E and F, the
error bars are percentile bootstrap 95% confidence intervals not
corrected for multiple comparisons. On panels B, C and D, the horizontal
and/or vertical segments are the estimates of the deciles (panels B and
C) or the quantiles (panel D, step of 0.05) of the distributions; the
thickest segments are the median estimates. On panel B, the diagonal
black line depicts the identity line. If any, significant results (based
on adjusted *P* values) in the shift (panel E) and difference
asymmetry (panel F) functions are highlighted using thick red circles. A
small pseudo-random movement has been added horizontally and vertically
to the raw data displayed on panel B to minimize the presence of points
fully overlapped. The estimates of the deciles of the marginal
distributions (panels B, C and D), the quantiles of the individual
differences (panel D), the decile differences (panel E) and the quantile
sums (panel F) have been computed using the Harrell-Davis estimator.

# 5 External regulation score

On panel A, the errors bars over the points are the standard deviations
around the means, while on panel D it is the percentile bootstrap 95%
confidence interval around the median estimate. On panels E and F, the
error bars are percentile bootstrap 95% confidence intervals not
corrected for multiple comparisons. On panels B, C and D, the horizontal
and/or vertical segments are the estimates of the deciles (panels B and
C) or the quantiles (panel D, step of 0.05) of the distributions; the
thickest segments are the median estimates. On panel B, the diagonal
black line depicts the identity line. If any, significant results (based
on adjusted *P* values) in the shift (panel E) and difference
asymmetry (panel F) functions are highlighted using thick red circles. A
small pseudo-random movement has been added horizontally and vertically
to the raw data displayed on panel B to minimize the presence of points
fully overlapped. The estimates of the deciles of the marginal
distributions (panels B, C and D), the quantiles of the individual
differences (panel D), the decile differences (panel E) and the quantile
sums (panel F) have been computed using the Harrell-Davis estimator.

# 6 Amotivation score

On panel A, the errors bars over the points are the standard deviations
around the means, while on panel D it is the percentile bootstrap 95%
confidence interval around the median estimate. On panels E and F, the
error bars are percentile bootstrap 95% confidence intervals not
corrected for multiple comparisons. On panels B, C and D, the horizontal
and/or vertical segments are the estimates of the deciles (panels B and
C) or the quantiles (panel D, step of 0.05) of the distributions; the
thickest segments are the median estimates. On panel B, the diagonal
black line depicts the identity line. If any, significant results (based
on adjusted *P* values) in the shift (panel E) and difference
asymmetry (panel F) functions are highlighted using thick red circles. A
small pseudo-random movement has been added horizontally and vertically
to the raw data displayed on panel B to minimize the presence of points
fully overlapped. The estimates of the deciles of the marginal
distributions (panels B, C and D), the quantiles of the individual
differences (panel D), the decile differences (panel E) and the quantile
sums (panel F) have been computed using the Harrell-Davis estimator.
