# Supplementary material for: Change in exercise capacity, physical activity and motivation for physical activity at 12 months after a cardiac rehabilitation program in coronary heart disease patients: a prospective, monocentric and observational study
Source: PeerJ. 2025 Feb 14;13:e18885. doi: 10.7717/peerj.18885 (PMC11831972; doi:10.7717/peerj.18885)
Supplement: Supplemental Information 5 [file peerj-13-18885-s005.html]

APA&Co project | SM5. Change in IPAQ-SF MET-min/week between 0 and 12 months


## Table of content

Code 

- Show All Code
- Hide All Code

# APA&Co project | SM5. Change in IPAQ-SF MET-min/week between 0 and 12 months

# 1 Descriptive statistics

```
targets::tar_read(DB_IPAQ_0_12) |>
  dplyr::select(-n_visits) |>  
  dplyr::group_by(MONTH) |> 
  skimr::skim()
```

Data summary

|  |  |
| --- | --- |
| Name | dplyr::group\_by(…) |
| Number of rows | 154 |
| Number of columns | 3 |
| \_\_\_\_\_\_\_\_\_\_\_\_\_\_\_\_\_\_\_\_\_\_\_ |  |
| Column type frequency: |  |
| factor | 1 |
| numeric | 1 |
| \_\_\_\_\_\_\_\_\_\_\_\_\_\_\_\_\_\_\_\_\_\_\_\_ |  |
| Group variables | MONTH |

**Variable type: factor**

| skim\_variable | MONTH | n\_missing | complete\_rate | ordered | n\_unique | top\_counts |
| --- | --- | --- | --- | --- | --- | --- |
| patient | 0 | 0 | 1 | FALSE | 77 | 1: 1, 2: 1, 3: 1, 4: 1 |
| patient | 12 | 0 | 1 | FALSE | 77 | 1: 1, 2: 1, 3: 1, 4: 1 |

**Variable type: numeric**

| skim\_variable | MONTH | n\_missing | complete\_rate | mean | sd | p0 | p25 | p50 | p75 | p100 | hist |
| --- | --- | --- | --- | --- | --- | --- | --- | --- | --- | --- | --- |
| MET\_MIN\_WK | 0 | 0 | 1 | 5222.97 | 3627.95 | 876 | 3144 | 4539 | 6396 | 27960 | ▇▃▁▁▁ |
| MET\_MIN\_WK | 12 | 0 | 1 | 4192.22 | 4722.84 | 0 | 1551 | 2796 | 5502 | 23106 | ▇▃▁▁▁ |

# 2 Estimate of the median of the individual changes

```
tar_read(change_IPAQ_0_12)$hd_pbci_diff |> 
  dplyr::filter(q == 0.5) |> 
  dplyr::mutate(dplyr::across(estimate:ci_u, ~round(.x, digits = 2)))
```

# 3 Shift and difference asymmetry functions

```
targets::tar_read(change_IPAQ_0_12)$p
```

Change in IPAQ-SF MET-min/week between 6 and 12 months (N = 77). On
panel A, the errors bars over the points are the standard deviations
around the means, while on panel D it is the percentile bootstrap 95%
confidence interval around the median estimate. On panels E and F, the
error bars are percentile bootstrap 95% confidence intervals not
corrected for multiple comparisons. On panels B, C and D, the horizontal
and/or vertical segments are the estimates of the deciles (panels B and
C) or the quantiles (panel D, step of 0.05) of the distributions; the
thickest segments are the median estimates. On panel B, the diagonal
black line depicts the identity line. If any, significant results (based
on adjusted p-values) in the shift (panel E) and difference asymmetry
(panel F) functions are highlighted using thick red circles. A small
pseudo-random movement has been added horizontally and vertically to the
raw data displayed on panel B to minimize the presence of points fully
overlapped. The estimates of the deciles of the marginal distributions
(panels B, C and D), the quantiles of the individual differences (panel
D), the decile differences (panel E) and the quantile sums (panel F)
have been computed using the Harrell-Davis estimator.
