# Supplementary material for: Change in exercise capacity, physical activity and motivation for physical activity at 12 months after a cardiac rehabilitation program in coronary heart disease patients: a prospective, monocentric and observational study
Source: PeerJ. 2025 Feb 14;13:e18885. doi: 10.7717/peerj.18885 (PMC11831972; doi:10.7717/peerj.18885)
Supplement: Supplemental Information 6 [file peerj-13-18885-s006.html]

APA&Co project | SM6. Data related to the shift and difference asymmetry functions to describe the change in 6MWT distance (0-12 months), IPAQ-SF MET-min/week (6-12 months; 0-12 months), and EMAPS scores (0-12 months)


## Table of content

Code 

- Show All Code
- Hide All Code

# APA&Co project | SM6. Data related to the shift and difference asymmetry functions to describe the change in 6MWT distance (0-12 months), IPAQ-SF MET-min/week (6-12 months; 0-12 months), and EMAPS scores (0-12 months)

```
res <-
  purrr::map2(list_titles, list_tables, function(x, y) {
  
  knitr::knit_child(text = c(
                    
                    "\n",
                    "# `r x`",
                    "\n",
                    "## Shift function",
                    "\n",
                    "```{r, echo = FALSE}",
                    "y$sf |> dplyr::mutate(
                    dplyr::across(`12`:ci_upper, ~round(.x, digits = 2)),
                    p_value = format(round(p_value, 3), nsmall = 3),
                    adj_p_value_bh = format(round(adj_p_value_bh, 3), nsmall = 3)
                    )",
                    "```",
                    "```{r, results='asis'}",
                    "cat(legend_sf)",
                    "```",
                    "\n",
                    "## Difference asymmetry function",
                    "\n",
                    "```{r, echo = FALSE}",
                    "y$daf |> dplyr::mutate(
                    dplyr::across(Est_q:ci.up, ~round(.x, digits = 2)),
                    p.value = format(round(p.value, 3), nsmall = 3),
                    adj_p_value_bh = format(round(adj_p_value_bh, 3), nsmall = 3)
                    )",
                    "```",
                    "```{r, results='asis'}",
                    "cat(legend_asym)",
                    "```"
  ),
  envir = environment(),
  quiet  = TRUE
  )
})

cat(unlist(res), sep = "\n")
```

# 1 6MWT distance (0-12 months)

## 1.1 Shift function

```
cat(legend_sf)
```

q = quantile; ci.low = lower bounds of the confidence intervals;
ci.up = upper bounds of the confidence intervals; adj\_p\_value\_bh = p
value adjusted for multiple comparisons using the Benjamini-Hochberg
False Discovery Rate method.

## 1.2 Difference asymmetry function

```
cat(legend_asym)
```

Est\_q = quantiles of differences; Est\_1.minus.q = 1 - quantiles of
differences; SUM = sum of quantiles; ci.low = lower bounds of the
confidence intervals; ci.up = upper bounds of the confidence intervals;
adj\_p\_value\_bh = p value adjusted for multiple comparisons using the
Benjamini-Hochberg False Discovery Rate method.

# 2 IPAQ-SF MET-min / week (6-12 months)

## 2.1 Shift function

```
cat(legend_sf)
```

q = quantile; ci.low = lower bounds of the confidence intervals;
ci.up = upper bounds of the confidence intervals; adj\_p\_value\_bh = p
value adjusted for multiple comparisons using the Benjamini-Hochberg
False Discovery Rate method.

## 2.2 Difference asymmetry function

```
cat(legend_asym)
```

Est\_q = quantiles of differences; Est\_1.minus.q = 1 - quantiles of
differences; SUM = sum of quantiles; ci.low = lower bounds of the
confidence intervals; ci.up = upper bounds of the confidence intervals;
adj\_p\_value\_bh = p value adjusted for multiple comparisons using the
Benjamini-Hochberg False Discovery Rate method.

# 3 IPAQ-SF MET-min / week (0-12 months)

## 3.1 Shift function

```
cat(legend_sf)
```

q = quantile; ci.low = lower bounds of the confidence intervals;
ci.up = upper bounds of the confidence intervals; adj\_p\_value\_bh = p
value adjusted for multiple comparisons using the Benjamini-Hochberg
False Discovery Rate method.

## 3.2 Difference asymmetry function

```
cat(legend_asym)
```

Est\_q = quantiles of differences; Est\_1.minus.q = 1 - quantiles of
differences; SUM = sum of quantiles; ci.low = lower bounds of the
confidence intervals; ci.up = upper bounds of the confidence intervals;
adj\_p\_value\_bh = p value adjusted for multiple comparisons using the
Benjamini-Hochberg False Discovery Rate method.

# 4 EMAPS | Intrinsic motivation (0-12 months)

## 4.1 Shift function

```
cat(legend_sf)
```

q = quantile; ci.low = lower bounds of the confidence intervals;
ci.up = upper bounds of the confidence intervals; adj\_p\_value\_bh = p
value adjusted for multiple comparisons using the Benjamini-Hochberg
False Discovery Rate method.

## 4.2 Difference asymmetry function

```
cat(legend_asym)
```

Est\_q = quantiles of differences; Est\_1.minus.q = 1 - quantiles of
differences; SUM = sum of quantiles; ci.low = lower bounds of the
confidence intervals; ci.up = upper bounds of the confidence intervals;
adj\_p\_value\_bh = p value adjusted for multiple comparisons using the
Benjamini-Hochberg False Discovery Rate method.

# 5 EMAPS | Integrated regulation (0-12 months)

## 5.1 Shift function

```
cat(legend_sf)
```

q = quantile; ci.low = lower bounds of the confidence intervals;
ci.up = upper bounds of the confidence intervals; adj\_p\_value\_bh = p
value adjusted for multiple comparisons using the Benjamini-Hochberg
False Discovery Rate method.

## 5.2 Difference asymmetry function

```
cat(legend_asym)
```

Est\_q = quantiles of differences; Est\_1.minus.q = 1 - quantiles of
differences; SUM = sum of quantiles; ci.low = lower bounds of the
confidence intervals; ci.up = upper bounds of the confidence intervals;
adj\_p\_value\_bh = p value adjusted for multiple comparisons using the
Benjamini-Hochberg False Discovery Rate method.

# 6 EMAPS | Identified regulation (0-12 months)

## 6.1 Shift function

```
cat(legend_sf)
```

q = quantile; ci.low = lower bounds of the confidence intervals;
ci.up = upper bounds of the confidence intervals; adj\_p\_value\_bh = p
value adjusted for multiple comparisons using the Benjamini-Hochberg
False Discovery Rate method.

## 6.2 Difference asymmetry function

```
cat(legend_asym)
```

Est\_q = quantiles of differences; Est\_1.minus.q = 1 - quantiles of
differences; SUM = sum of quantiles; ci.low = lower bounds of the
confidence intervals; ci.up = upper bounds of the confidence intervals;
adj\_p\_value\_bh = p value adjusted for multiple comparisons using the
Benjamini-Hochberg False Discovery Rate method.

# 7 EMAPS | Introjected regulation (0-12 months)

## 7.1 Shift function

```
cat(legend_sf)
```

q = quantile; ci.low = lower bounds of the confidence intervals;
ci.up = upper bounds of the confidence intervals; adj\_p\_value\_bh = p
value adjusted for multiple comparisons using the Benjamini-Hochberg
False Discovery Rate method.

## 7.2 Difference asymmetry function

```
cat(legend_asym)
```

Est\_q = quantiles of differences; Est\_1.minus.q = 1 - quantiles of
differences; SUM = sum of quantiles; ci.low = lower bounds of the
confidence intervals; ci.up = upper bounds of the confidence intervals;
adj\_p\_value\_bh = p value adjusted for multiple comparisons using the
Benjamini-Hochberg False Discovery Rate method.

# 8 EMAPS | External regulation (0-12 months)

## 8.1 Shift function

```
cat(legend_sf)
```

q = quantile; ci.low = lower bounds of the confidence intervals;
ci.up = upper bounds of the confidence intervals; adj\_p\_value\_bh = p
value adjusted for multiple comparisons using the Benjamini-Hochberg
False Discovery Rate method.

## 8.2 Difference asymmetry function

```
cat(legend_asym)
```

Est\_q = quantiles of differences; Est\_1.minus.q = 1 - quantiles of
differences; SUM = sum of quantiles; ci.low = lower bounds of the
confidence intervals; ci.up = upper bounds of the confidence intervals;
adj\_p\_value\_bh = p value adjusted for multiple comparisons using the
Benjamini-Hochberg False Discovery Rate method.

# 9 EMAPS | Amotivation (0-12 months)

## 9.1 Shift function

```
cat(legend_sf)
```

q = quantile; ci.low = lower bounds of the confidence intervals;
ci.up = upper bounds of the confidence intervals; adj\_p\_value\_bh = p
value adjusted for multiple comparisons using the Benjamini-Hochberg
False Discovery Rate method.

## 9.2 Difference asymmetry function

```
cat(legend_asym)
```

Est\_q = quantiles of differences; Est\_1.minus.q = 1 - quantiles of
differences; SUM = sum of quantiles; ci.low = lower bounds of the
confidence intervals; ci.up = upper bounds of the confidence intervals;
adj\_p\_value\_bh = p value adjusted for multiple comparisons using the
Benjamini-Hochberg False Discovery Rate method.
