# Supplementary material for: Change in exercise capacity, physical activity and motivation for physical activity at 12 months after a cardiac rehabilitation program in coronary heart disease patients: a prospective, monocentric and observational study
Source: PeerJ. 2025 Feb 14;13:e18885. doi: 10.7717/peerj.18885 (PMC11831972; doi:10.7717/peerj.18885)
Supplement: Supplemental Information 7 [file peerj-13-18885-s007.html]

APA&Co project | SM7. Characteristics of the participants with missing data at 12 months


## Table of content

Code 

- Show All Code
- Hide All Code

# APA&Co project | SM7. Characteristics of the participants with missing data at 12 months

# 1 Six-min walking test distance

```
# Get IDs with missing data
ids_6MWT_missing <- extract_missing_ids(data1 = db_6MWT_init, data2 = db_6MWT_final)
db_6MWT_init$na_status <- ifelse(db_6MWT_init$patient %in% ids_6MWT_missing, "Incomplete", "Complete")
db_6MWT_init$na_status <- factor(db_6MWT_init$na_status, levels = c("Incomplete", "Complete"))

# Show descriptive statistics
INCLUSION_cleaned[INCLUSION_cleaned$patient %in% ids_6MWT_missing, ] |> 
  skimr::skim() |> 
  as.data.frame()
```

```
ggplot(data = db_6MWT_init |>  dplyr::filter(MONTH == "0"), aes(x = "", y = DIST_M, color = na_status)) +
  geom_boxplot(position = position_dodge(width = 0.75), width = 0.25, outlier.size = 0) +
  geom_point(position = position_jitterdodge(jitter.width = 0.05, dodge.width = 0.75)) +
  coord_flip() +
  scale_color_manual(values = c("red", "blue"), guide = guide_legend(reverse = TRUE)) +
  labs(title = "6MWT", x = NULL, y = "6-min walking distance (m)", color = "Data completness status") +
  theme(axis.ticks.y = element_blank())
```

## 1.1 IPAQ-SF MET-min/week

```
# Get IDs with missing data
ids_IPAQ_missing <- extract_missing_ids(data1 = db_IPAQ_init, data2 = db_IPAQ_final, MONTH = "6")
db_IPAQ_init$na_status <- ifelse(db_IPAQ_init$patient %in% ids_IPAQ_missing, "Incomplete", "Complete")
db_IPAQ_init$na_status <- factor(db_IPAQ_init$na_status, levels = c("Incomplete", "Complete"))

# Show descriptive statistics
INCLUSION_cleaned[INCLUSION_cleaned$patient %in% ids_IPAQ_missing,] |> 
  skimr::skim() |> 
  as.data.frame()
```

```
ggplot(data = db_IPAQ_init |>  dplyr::filter(MONTH == "0"), aes(x = "", y = MET_MIN_WK, color = na_status)) +
  geom_boxplot(position = position_dodge(width = 0.75), width = 0.25, outlier.size = 0) +
  geom_point(position = position_jitterdodge(jitter.width = 0.05, dodge.width = 0.75)) +
  coord_flip() +
  scale_color_manual(values = c("red", "blue"), guide = guide_legend(reverse = TRUE)) +
  labs(title = "IPAQ-SF", x = NULL, y = "IPAQ-SF (MET-min/week)", color = "Data completness status") +
  theme(axis.ticks.y = element_blank())
```

## 1.2 EMAPS

```
# Get IDs with missing data
ids_EMAPS_missing <- extract_missing_ids(data1 = db_EMAPS_init, data2 = db_EMAPS_final)
db_EMAPS_init$na_status <- ifelse(db_EMAPS_init$patient %in% ids_EMAPS_missing, "Incomplete", "Complete")
db_EMAPS_init$na_status <- factor(db_EMAPS_init$na_status, levels = c("Incomplete", "Complete"))

# Show descriptive statistics
INCLUSION_cleaned[INCLUSION_cleaned$patient %in% ids_EMAPS_missing,] |> 
  skimr::skim() |> 
  as.data.frame()
```

```
ggplot(data = db_EMAPS_init |>  
         dplyr::filter(MONTH == "0") |> 
         tidyr::pivot_longer(
           cols = c(INTRINSIC:AMOTIVATION), 
           names_to = "type_motivation", 
           values_to = "Score"
           ) |> 
         dplyr::mutate(type_motivation = factor(type_motivation, levels = c("INTRINSIC", "INTEGRATED", "IDENTIFIED", "INTROJECTED", "EXTERNAL", "AMOTIVATION"))), 
       
       aes(x = "", y = Score, color = na_status)) +
  geom_boxplot(position = position_dodge(width = 0.75), width = 0.25, outlier.size = 0) +
  geom_point(position = position_jitterdodge(jitter.width = 0.05, dodge.width = 0.75)) +
  coord_flip() +
  scale_color_manual(values = c("red", "blue"), guide = guide_legend(reverse = TRUE)) +
  labs(title = "EMAPS", x = NULL, color = "Data completness status") +
  theme(axis.ticks.y = element_blank()) +
  facet_wrap(~ type_motivation)
```
