# Supplementary material for: Change in exercise capacity, physical activity and motivation for physical activity at 12 months after a cardiac rehabilitation program in coronary heart disease patients: a prospective, monocentric and observational study
Source: PeerJ. 2025 Feb 14;13:e18885. doi: 10.7717/peerj.18885 (PMC11831972; doi:10.7717/peerj.18885)
Supplement: Supplemental Information 8 [file peerj-13-18885-s008.html]

APA&Co project | SM8. Figures to show the changes in the EMAPS scores associated to the different scnearii of change in the motivational profile between 0 and 12 months after the CR program


## Table of content

Code 

- Show All Code
- Hide All Code

# APA&Co project | SM8. Figures to show the changes in the EMAPS scores associated to the different scnearii of change in the motivational profile between 0 and 12 months after the CR program

# 1 Visualize the EMAPS score deltas per motivation variable depending on the profile transition

```
targets::tar_read(p_DB_EMAPS_0_12_diffs)
```

# 2 Visualize the whole profile of EMAPS score deltas depending on the profile transition

```
targets::tar_read(p_DB_EMAPS_0_12_diffs_spag)
```
