# Supplementary material for: Change in exercise capacity, physical activity and motivation for physical activity at 12 months after a cardiac rehabilitation program in coronary heart disease patients: a prospective, monocentric and observational study
Source: PeerJ. 2025 Feb 14;13:e18885. doi: 10.7717/peerj.18885 (PMC11831972; doi:10.7717/peerj.18885)
Supplement: Supplemental Information 9 [file peerj-13-18885-s009.html]

APA&Co project | SM9. Models to analyse the classes of changes in 6MWT distance and IPAQ-SF MET-min/week and their potential predictors


## Table of content

Code 

- Show All Code
- Hide All Code

# APA&Co project | SM9. Models to analyse the classes of changes in 6MWT distance and IPAQ-SF MET-min/week and their potential predictors

```
# Load all models and related metrics
## 6MWT models
tar_load(model_6MWT_n1)
tar_load(model_6MWT_n2)
tar_load(model_6MWT_n3)
tar_load(model_6MWT_n4)
tar_load(model_6MWT_n5)
tar_load(compa_latent_mixed_models_table_6MWT)
tar_load(predictors_6MWT_classes)

## IPAQ-SF models
tar_load(model_IPAQ_n1)
tar_load(model_IPAQ_n2)
tar_load(model_IPAQ_n3)
tar_load(model_IPAQ_n4)
tar_load(model_IPAQ_n5)
tar_load(compa_latent_mixed_models_table_IPAQ)
tar_load(predictors_IPAQ_classes)
```

# 1 6MWT models

## 1.1 Latent class mixed models

```
 bind_rows(
  res_model_6MWT_n1,
  res_model_6MWT_n2,
  res_model_6MWT_n3,
  res_model_6MWT_n4,
  res_model_6MWT_n5
) |> 
  select(model, everything()) |>
  left_join(
    compa_latent_mixed_models_table_6MWT |> 
      as.data.frame() |>  
      tibble::rownames_to_column(var = "model")
  ) |> 
  mutate(
    model = forcats::fct_recode(
      model,
      "6MWT | 1-class" = "model_6MWT_n1",
      "6MWT | 2-class" = "model_6MWT_n2",
      "6MWT | 3-class" = "model_6MWT_n3",
      "6MWT | 4-class" = "model_6MWT_n4",
      "6MWT | 5-class" = "model_6MWT_n5"
    ),
    `p-value` = ifelse(
      `p-value` < 0.001,
      "< 0.001",
      format(janitor::round_half_up(`p-value`, 3), nsmall = 3)
    ),
    across(c(AIC:`%class5`), ~  format(janitor::round_half_up(.x, 2), nsmall = 2))
  ) |>
  select(-c(Se, Wald)) |> 
  rename(
   Model = model,
   Term = coef_name,
   Coefficient = coef,
   Entropy = entropy,
   P = "p-value"
  ) |> 
  reactable::reactable(
      columns = list(Model = reactable::colDef(
        style = reactable::JS(
          "function(rowInfo, colInfo, state) {
        var firstSorted = state.sorted[0]
        // Merge cells if unsorted or sorting by Model
        if (!firstSorted || firstSorted.id === 'Model') {
          var prevRow = state.pageRows[rowInfo.viewIndex - 1]
          if (prevRow && rowInfo.row['Model'] === prevRow['Model']) {
            return { visibility: 'hidden' }
          }
        }
      }"
        )
      ),
      AIC = reactable::colDef(
        style = reactable::JS(
          "function(rowInfo, colInfo, state) {
        var firstSorted = state.sorted[0]
        // Merge cells if unsorted or sorting by AIC
        if (!firstSorted || firstSorted.id === 'AIC') {
          var prevRow = state.pageRows[rowInfo.viewIndex - 1]
          if (prevRow && rowInfo.row['AIC'] === prevRow['AIC']) {
            return { visibility: 'hidden' }
          }
        }
      }"
        )
      ),
      BIC = reactable::colDef(
        style = reactable::JS(
          "function(rowInfo, colInfo, state) {
        var firstSorted = state.sorted[0]
        // Merge cells if unsorted or sorting by BIC
        if (!firstSorted || firstSorted.id === 'BIC') {
          var prevRow = state.pageRows[rowInfo.viewIndex - 1]
          if (prevRow && rowInfo.row['BIC'] === prevRow['BIC']) {
            return { visibility: 'hidden' }
          }
        }
      }"
        )
      ),
      Entropy = reactable::colDef(
        style = reactable::JS(
          "function(rowInfo, colInfo, state) {
        var firstSorted = state.sorted[0]
        // Merge cells if unsorted or sorting by Entropy
        if (!firstSorted || firstSorted.id === 'Entropy') {
          var prevRow = state.pageRows[rowInfo.viewIndex - 1]
          if (prevRow && rowInfo.row['Entropy'] === prevRow['Entropy']) {
            return { visibility: 'hidden' }
          }
        }
      }"
        )
      ),
      `%class1` = reactable::colDef(
        style = reactable::JS(
          "function(rowInfo, colInfo, state) {
        var firstSorted = state.sorted[0]
        // Merge cells if unsorted or sorting by %class1
        if (!firstSorted || firstSorted.id === '%class1') {
          var prevRow = state.pageRows[rowInfo.viewIndex - 1]
          if (prevRow && rowInfo.row['%class1'] === prevRow['%class1']) {
            return { visibility: 'hidden' }
          }
        }
      }"
        )
      ),
      `%class2` = reactable::colDef(
        style = reactable::JS(
          "function(rowInfo, colInfo, state) {
        var firstSorted = state.sorted[0]
        // Merge cells if unsorted or sorting by %class2
        if (!firstSorted || firstSorted.id === '%class2') {
          var prevRow = state.pageRows[rowInfo.viewIndex - 1]
          if (prevRow && rowInfo.row['%class2'] === prevRow['%class2']) {
            return { visibility: 'hidden' }
          }
        }
      }"
        )
      ),
      `%class3` = reactable::colDef(
        style = reactable::JS(
          "function(rowInfo, colInfo, state) {
        var firstSorted = state.sorted[0]
        // Merge cells if unsorted or sorting by %class3
        if (!firstSorted || firstSorted.id === '%class3') {
          var prevRow = state.pageRows[rowInfo.viewIndex - 1]
          if (prevRow && rowInfo.row['%class3'] === prevRow['%class3']) {
            return { visibility: 'hidden' }
          }
        }
      }"
        )
      ),
      `%class4` = reactable::colDef(
        style = reactable::JS(
          "function(rowInfo, colInfo, state) {
        var firstSorted = state.sorted[0]
        // Merge cells if unsorted or sorting by %class4
        if (!firstSorted || firstSorted.id === '%class4') {
          var prevRow = state.pageRows[rowInfo.viewIndex - 1]
          if (prevRow && rowInfo.row['%class4'] === prevRow['%class4']) {
            return { visibility: 'hidden' }
          }
        }
      }"
        )
      ),
      `%class5` = reactable::colDef(
        style = reactable::JS(
          "function(rowInfo, colInfo, state) {
        var firstSorted = state.sorted[0]
        // Merge cells if unsorted or sorting by %class5
        if (!firstSorted || firstSorted.id === '%class5') {
          var prevRow = state.pageRows[rowInfo.viewIndex - 1]
          if (prevRow && rowInfo.row['%class5'] === prevRow['%class5']) {
            return { visibility: 'hidden' }
          }
        }
      }"
        )
      )
      ),
      outlined = TRUE,
      striped = TRUE,
      defaultPageSize = 30
    )
```

## 1.2 Multinomial regression model (based on the 3-class latent mixed model)

```
res_model_preds_6MWT |> 
  select(model, everything()) |>
  mutate(
    model = forcats::fct_recode(
      model,
      "6MWT | 3-class" = "predictors_6MWT_classes"
    ),
    `p-value` = ifelse(
      as.numeric(`p-value`) < 0.001,
      "< 0.001",
      format(janitor::round_half_up(as.numeric(`p-value`), 3), nsmall = 3)
    )
  ) |>
  select(-c(`    Se**`, `  Wald`)) |> 
  rename(
   Model = model,
   Term = coef_name,
   Coefficient = "     coef",
   P = "p-value"
  ) |> 
  reactable::reactable(
      columns = list(Model = reactable::colDef(
        style = reactable::JS(
          "function(rowInfo, colInfo, state) {
        var firstSorted = state.sorted[0]
        // Merge cells if unsorted or sorting by Model
        if (!firstSorted || firstSorted.id === 'Model') {
          var prevRow = state.pageRows[rowInfo.viewIndex - 1]
          if (prevRow && rowInfo.row['Model'] === prevRow['Model']) {
            return { visibility: 'hidden' }
          }
        }
      }"
        )
      )),
      outlined = TRUE,
      striped = TRUE,
      defaultPageSize = 30
    )
```

# 2 IPAQ-SF models

## 2.1 Latent class mixed models

```
bind_rows(
  res_model_IPAQ_n1,
  res_model_IPAQ_n2,
  res_model_IPAQ_n3,
  res_model_IPAQ_n4,
  res_model_IPAQ_n5
) |> 
  select(model, everything()) |>
  left_join(
    compa_latent_mixed_models_table_IPAQ |> 
      as.data.frame() |>  
      tibble::rownames_to_column(var = "model")
  ) |> 
  mutate(
    model = forcats::fct_recode(
      model,
      "IPAQ | 1-class" = "model_IPAQ_n1",
      "IPAQ | 2-class" = "model_IPAQ_n2",
      "IPAQ | 3-class" = "model_IPAQ_n3",
    ),
    `p-value` = ifelse(
      `p-value` < 0.001,
      "< 0.001",
      format(janitor::round_half_up(`p-value`, 3), nsmall = 3)
    ),
    across(c(AIC:`%class5`), ~  format(janitor::round_half_up(.x, 2), nsmall = 2))
  ) |>
  select(-c(Se, Wald)) |> 
  rename(
   Model = model,
   Term = coef_name,
   Coefficient = coef,
   Entropy = entropy,
   P = "p-value"
  ) |> 
  reactable::reactable(
      columns = list(Model = reactable::colDef(
        style = reactable::JS(
          "function(rowInfo, colInfo, state) {
        var firstSorted = state.sorted[0]
        // Merge cells if unsorted or sorting by Model
        if (!firstSorted || firstSorted.id === 'Model') {
          var prevRow = state.pageRows[rowInfo.viewIndex - 1]
          if (prevRow && rowInfo.row['Model'] === prevRow['Model']) {
            return { visibility: 'hidden' }
          }
        }
      }"
        )
      ),
      AIC = reactable::colDef(
        style = reactable::JS(
          "function(rowInfo, colInfo, state) {
        var firstSorted = state.sorted[0]
        // Merge cells if unsorted or sorting by AIC
        if (!firstSorted || firstSorted.id === 'AIC') {
          var prevRow = state.pageRows[rowInfo.viewIndex - 1]
          if (prevRow && rowInfo.row['AIC'] === prevRow['AIC']) {
            return { visibility: 'hidden' }
          }
        }
      }"
        )
      ),
      BIC = reactable::colDef(
        style = reactable::JS(
          "function(rowInfo, colInfo, state) {
        var firstSorted = state.sorted[0]
        // Merge cells if unsorted or sorting by BIC
        if (!firstSorted || firstSorted.id === 'BIC') {
          var prevRow = state.pageRows[rowInfo.viewIndex - 1]
          if (prevRow && rowInfo.row['BIC'] === prevRow['BIC']) {
            return { visibility: 'hidden' }
          }
        }
      }"
        )
      ),
      Entropy = reactable::colDef(
        style = reactable::JS(
          "function(rowInfo, colInfo, state) {
        var firstSorted = state.sorted[0]
        // Merge cells if unsorted or sorting by Entropy
        if (!firstSorted || firstSorted.id === 'Entropy') {
          var prevRow = state.pageRows[rowInfo.viewIndex - 1]
          if (prevRow && rowInfo.row['Entropy'] === prevRow['Entropy']) {
            return { visibility: 'hidden' }
          }
        }
      }"
        )
      ),
      `%class1` = reactable::colDef(
        style = reactable::JS(
          "function(rowInfo, colInfo, state) {
        var firstSorted = state.sorted[0]
        // Merge cells if unsorted or sorting by %class1
        if (!firstSorted || firstSorted.id === '%class1') {
          var prevRow = state.pageRows[rowInfo.viewIndex - 1]
          if (prevRow && rowInfo.row['%class1'] === prevRow['%class1']) {
            return { visibility: 'hidden' }
          }
        }
      }"
        )
      ),
      `%class2` = reactable::colDef(
        style = reactable::JS(
          "function(rowInfo, colInfo, state) {
        var firstSorted = state.sorted[0]
        // Merge cells if unsorted or sorting by %class2
        if (!firstSorted || firstSorted.id === '%class2') {
          var prevRow = state.pageRows[rowInfo.viewIndex - 1]
          if (prevRow && rowInfo.row['%class2'] === prevRow['%class2']) {
            return { visibility: 'hidden' }
          }
        }
      }"
        )
      ),
      `%class3` = reactable::colDef(
        style = reactable::JS(
          "function(rowInfo, colInfo, state) {
        var firstSorted = state.sorted[0]
        // Merge cells if unsorted or sorting by %class3
        if (!firstSorted || firstSorted.id === '%class3') {
          var prevRow = state.pageRows[rowInfo.viewIndex - 1]
          if (prevRow && rowInfo.row['%class3'] === prevRow['%class3']) {
            return { visibility: 'hidden' }
          }
        }
      }"
        )
      )
      ),
      outlined = TRUE,
      striped = TRUE,
      defaultPageSize = 30
    )
```

## 2.2 Multinomial regression model (based on the 2-class latent mixed model)

```
res_model_preds_IPAQ |> 
  select(model, everything()) |>
  mutate(
    model = forcats::fct_recode(
      model,
      "IPAQ | 2-class" = "predictors_IPAQ_classes"
    ),
    `p-value` = ifelse(
      as.numeric(`p-value`) < 0.001,
      "< 0.001",
      format(janitor::round_half_up(as.numeric(`p-value`), 3), nsmall = 3)
    )
  ) |>
  select(-c(`   Se**`, `  Wald`)) |> 
  rename(
   Model = model,
   Term = coef_name,
   Coefficient = "    coef",
   P = "p-value"
  ) |> 
  reactable::reactable(
      columns = list(Model = reactable::colDef(
        style = reactable::JS(
          "function(rowInfo, colInfo, state) {
        var firstSorted = state.sorted[0]
        // Merge cells if unsorted or sorting by Model
        if (!firstSorted || firstSorted.id === 'Model') {
          var prevRow = state.pageRows[rowInfo.viewIndex - 1]
          if (prevRow && rowInfo.row['Model'] === prevRow['Model']) {
            return { visibility: 'hidden' }
          }
        }
      }"
        )
      )),
      outlined = TRUE,
      striped = TRUE,
      defaultPageSize = 30
    )
```
