# Supplementary material for: Change in exercise capacity, physical activity and motivation for physical activity at 12 months after a cardiac rehabilitation program in coronary heart disease patients: a prospective, monocentric and observational study
Source: PeerJ. 2025 Feb 14;13:e18885. doi: 10.7717/peerj.18885 (PMC11831972; doi:10.7717/peerj.18885)
Supplement: Supplemental Information 10 [file peerj-13-18885-s010.html]

APA&Co project | Results shown in the manuscript


## Table of content

Code 

- Show All Code
- Hide All Code

# APA&Co project | Results shown in the manuscript

# 1 Descriptive analysis of the participants

## 1.1 Overview

```
targets::tar_read(analysis_INCLUSION)
```

## 1.2 Sex (1 = men; 2 = women)

```
targets::tar_read(analysis_sex)
```

## 1.3 Age

```
targets::tar_read(analysis_age)
```

## 1.4 Height

```
targets::tar_read(analysis_height)
```

## 1.5 Weight

```
targets::tar_read(analysis_weight)
```

## 1.6 Body mass index

```
targets::tar_read(analysis_bmi)
```

## 1.7 Angioplasty (0 = no angioplasty; 1 = with angioplasty)

```
targets::tar_read(analysis_angioplasty)[, c(1:2)]
```

## 1.8 Bypass (0 = no bypass; 1 = with bypass)

```
targets::tar_read(analysis_bypass)[, c(1:2)]
```

# 2 Change in 6MWT distance between 0 and 12 months

## 2.1 Descriptive statistics

```
targets::tar_read(DB_6MWT_0_12) |> 
  dplyr::select(-n_visits) |>   
  dplyr::group_by(MONTH) |> 
  skimr::skim()
```

Data summary

|  |  |
| --- | --- |
| Name | dplyr::group\_by(�) |
| Number of rows | 150 |
| Number of columns | 3 |
| \_\_\_\_\_\_\_\_\_\_\_\_\_\_\_\_\_\_\_\_\_\_\_ |  |
| Column type frequency: |  |
| factor | 1 |
| numeric | 1 |
| \_\_\_\_\_\_\_\_\_\_\_\_\_\_\_\_\_\_\_\_\_\_\_\_ |  |
| Group variables | MONTH |

**Variable type: factor**

| skim\_variable | MONTH | n\_missing | complete\_rate | ordered | n\_unique | top\_counts |
| --- | --- | --- | --- | --- | --- | --- |
| patient | 0 | 0 | 1 | FALSE | 75 | 1: 1, 2: 1, 3: 1, 4: 1 |
| patient | 12 | 0 | 1 | FALSE | 75 | 1: 1, 2: 1, 3: 1, 4: 1 |

**Variable type: numeric**

| skim\_variable | MONTH | n\_missing | complete\_rate | mean | sd | p0 | p25 | p50 | p75 | p100 | hist |
| --- | --- | --- | --- | --- | --- | --- | --- | --- | --- | --- | --- |
| DIST\_M | 0 | 0 | 1 | 605.44 | 71.96 | 411 | 559 | 603 | 636 | 816 | ????? |
| DIST\_M | 12 | 0 | 1 | 618.00 | 81.49 | 400 | 574 | 622 | 652 | 880 | ????? |

## 2.2 T-test

```
targets::tar_read(t_test_results_6MWT)
```

```
## 
##  Paired t-test
## 
## data:  DIST_M by MONTH
## t = 2.8614, df = 74, p-value = 0.005481
## alternative hypothesis: true mean difference is not equal to 0
## 95 percent confidence interval:
##   3.813784 21.306216
## sample estimates:
## mean difference 
##           12.56
```

## 2.3 Pearson correlation

```
# Rearrange data
data_6MWT <- 
  targets::tar_read(DB_6MWT_0_12) |> 
  tidyr::pivot_wider(names_from = MONTH, values_from = DIST_M)

# Compute Pearson correlation
cor.test(data_6MWT$`0`, data_6MWT$`12`)
```

```
## 
##  Pearson's product-moment correlation
## 
## data:  data_6MWT$`0` and data_6MWT$`12`
## t = 16.202, df = 73, p-value < 2.2e-16
## alternative hypothesis: true correlation is not equal to 0
## 95 percent confidence interval:
##  0.8227583 0.9256709
## sample estimates:
##       cor 
## 0.8845449
```

## 2.4 Effect size (dav)

```
mean_diff <- mean(data_6MWT$`12` - data_6MWT$`0`)
sd1 <- sd(data_6MWT$`0`)
sd2 <- sd(data_6MWT$`12`)
dav <- mean_diff / sqrt(((sd1^2 + sd2^2) / 2))
round(dav, 2)
```

```
## [1] 0.16
```

## 2.5 Estimate of the median of the individual changes

```
tar_read(change_6MWT)$hd_pbci_diff |> 
  dplyr::filter(q == 0.5)  |> 
  dplyr::mutate(dplyr::across(estimate:ci_u, ~round(.x, digits = 2)))
```

## 2.6 Shift and difference asymmetry functions

```
targets::tar_read(change_6MWT)$p
```

Change in 6-min walking test (6MWT) distance between 0 and 12 months (N
= 75). On panel A, the errors bars over the points are the standard
deviations around the means, while on panel D it is the percentile
bootstrap 95% confidence interval around the median estimate. On panels
E and F, the error bars are percentile bootstrap 95% confidence
intervals not corrected for multiple comparisons. On panels B, C and D,
the horizontal and/or vertical segments are the estimates of the deciles
(panels B and C) or the quantiles (panel D, step of 0.05) of the
distributions; the thickest segments are the median estimates. On panel
B, the diagonal black line depicts the identity line. If any,
significant results (based on adjusted p-values) in the shift (panel E)
and difference asymmetry (panel F) functions are highlighted using thick
red circles. A small pseudo-random movement has been added horizontally
and vertically to the raw data displayed on panel B to minimize the
presence of points fully overlapped. The estimates of the deciles of the
marginal distributions (panels B, C and D), the quantiles of the
individual differences (panel D), the decile differences (panel E) and
the quantile sums (panel F) have been computed using the Harrell-Davis
estimator.

# 3 Change in IPAQ-SF MET-min/week between 6 and 12 months

## 3.1 Descriptive statistics

```
targets::tar_read(DB_IPAQ_6_12) |>
  dplyr::select(-n_visits) |>  
  dplyr::group_by(MONTH) |> 
  skimr::skim()
```

Data summary

|  |  |
| --- | --- |
| Name | dplyr::group\_by(�) |
| Number of rows | 154 |
| Number of columns | 3 |
| \_\_\_\_\_\_\_\_\_\_\_\_\_\_\_\_\_\_\_\_\_\_\_ |  |
| Column type frequency: |  |
| factor | 1 |
| numeric | 1 |
| \_\_\_\_\_\_\_\_\_\_\_\_\_\_\_\_\_\_\_\_\_\_\_\_ |  |
| Group variables | MONTH |

**Variable type: factor**

| skim\_variable | MONTH | n\_missing | complete\_rate | ordered | n\_unique | top\_counts |
| --- | --- | --- | --- | --- | --- | --- |
| patient | 6 | 0 | 1 | FALSE | 77 | 1: 1, 2: 1, 3: 1, 4: 1 |
| patient | 12 | 0 | 1 | FALSE | 77 | 1: 1, 2: 1, 3: 1, 4: 1 |

**Variable type: numeric**

| skim\_variable | MONTH | n\_missing | complete\_rate | mean | sd | p0 | p25 | p50 | p75 | p100 | hist |
| --- | --- | --- | --- | --- | --- | --- | --- | --- | --- | --- | --- |
| MET\_MIN\_WK | 6 | 0 | 1 | 3999.91 | 3588.04 | 0 | 1680 | 3318 | 4914 | 20040 | ????? |
| MET\_MIN\_WK | 12 | 0 | 1 | 4192.22 | 4722.84 | 0 | 1551 | 2796 | 5502 | 23106 | ????? |

## 3.2 Estimate of the median of the individual changes

```
tar_read(change_IPAQ_6_12)$hd_pbci_diff |> 
  dplyr::filter(q == 0.5) |> 
  dplyr::mutate(dplyr::across(estimate:ci_u, ~round(.x, digits = 2)))
```

## 3.3 Shift and difference asymmetry functions

```
targets::tar_read(change_IPAQ_6_12)$p
```

Change in IPAQ-SF MET-min/week between 6 and 12 months (N = 77). On
panel A, the errors bars over the points are the standard deviations
around the means, while on panel D it is the percentile bootstrap 95%
confidence interval around the median estimate. On panels E and F, the
error bars are percentile bootstrap 95% confidence intervals not
corrected for multiple comparisons. On panels B, C and D, the horizontal
and/or vertical segments are the estimates of the deciles (panels B and
C) or the quantiles (panel D, step of 0.05) of the distributions; the
thickest segments are the median estimates. On panel B, the diagonal
black line depicts the identity line. If any, significant results (based
on adjusted p-values) in the shift (panel E) and difference asymmetry
(panel F) functions are highlighted using thick red circles. A small
pseudo-random movement has been added horizontally and vertically to the
raw data displayed on panel B to minimize the presence of points fully
overlapped. The estimates of the deciles of the marginal distributions
(panels B, C and D), the quantiles of the individual differences (panel
D), the decile differences (panel E) and the quantile sums (panel F)
have been computed using the Harrell-Davis estimator.

# 4 Change in EMAPS scores between 0 and 12 months

## 4.1 Descriptive statistics

```
targets::tar_read(DB_EMAPS_0_12) |> 
  dplyr::select(-n_visits) |>  
  dplyr::group_by(MONTH) |> 
  skimr::skim()
```

Data summary

|  |  |
| --- | --- |
| Name | dplyr::group\_by(�) |
| Number of rows | 152 |
| Number of columns | 8 |
| \_\_\_\_\_\_\_\_\_\_\_\_\_\_\_\_\_\_\_\_\_\_\_ |  |
| Column type frequency: |  |
| factor | 1 |
| numeric | 6 |
| \_\_\_\_\_\_\_\_\_\_\_\_\_\_\_\_\_\_\_\_\_\_\_\_ |  |
| Group variables | MONTH |

**Variable type: factor**

| skim\_variable | MONTH | n\_missing | complete\_rate | ordered | n\_unique | top\_counts |
| --- | --- | --- | --- | --- | --- | --- |
| patient | 0 | 0 | 1 | FALSE | 76 | 1: 1, 2: 1, 3: 1, 4: 1 |
| patient | 12 | 0 | 1 | FALSE | 76 | 1: 1, 2: 1, 3: 1, 4: 1 |

**Variable type: numeric**

| skim\_variable | MONTH | n\_missing | complete\_rate | mean | sd | p0 | p25 | p50 | p75 | p100 | hist |
| --- | --- | --- | --- | --- | --- | --- | --- | --- | --- | --- | --- |
| INTRINSIC | 0 | 0 | 1 | 5.61 | 0.87 | 3.00 | 5.00 | 5.67 | 6.00 | 7.00 | ????? |
| INTRINSIC | 12 | 0 | 1 | 5.60 | 1.29 | 2.00 | 5.25 | 6.00 | 6.33 | 7.00 | ????? |
| INTEGRATED | 0 | 0 | 1 | 4.95 | 1.46 | 1.33 | 4.00 | 5.33 | 6.00 | 7.00 | ????? |
| INTEGRATED | 12 | 0 | 1 | 5.24 | 1.58 | 1.00 | 4.58 | 5.67 | 6.33 | 7.00 | ????? |
| IDENTIFIED | 0 | 0 | 1 | 6.02 | 0.76 | 3.67 | 5.67 | 6.00 | 6.67 | 7.00 | ????? |
| IDENTIFIED | 12 | 0 | 1 | 6.00 | 1.09 | 2.33 | 5.67 | 6.33 | 6.75 | 7.00 | ????? |
| INTROJECTED | 0 | 0 | 1 | 4.39 | 1.24 | 2.00 | 3.58 | 4.17 | 5.33 | 7.00 | ????? |
| INTROJECTED | 12 | 0 | 1 | 4.67 | 1.54 | 1.00 | 3.33 | 5.00 | 5.75 | 7.00 | ????? |
| EXTERNAL | 0 | 0 | 1 | 1.49 | 0.77 | 1.00 | 1.00 | 1.00 | 1.67 | 5.33 | ????? |
| EXTERNAL | 12 | 0 | 1 | 1.34 | 0.66 | 1.00 | 1.00 | 1.00 | 1.33 | 3.67 | ????? |
| AMOTIVATION | 0 | 0 | 1 | 1.48 | 1.00 | 1.00 | 1.00 | 1.00 | 1.33 | 6.00 | ????? |
| AMOTIVATION | 12 | 0 | 1 | 1.17 | 0.55 | 1.00 | 1.00 | 1.00 | 1.00 | 4.00 | ????? |

## 4.2 Estimates of the medians of the individual changes

```
targets::tar_load(change_EMAPS)
res <-
  purrr::map(change_EMAPS, function(x) {
  
  knitr::knit_child(text = c(
                    
                    "\n",
                    "### `r x$variable`",
                    "\n",
                    "```{r, echo = FALSE}",
                    "x$hd_pbci_diff |>  
                     dplyr::filter(q == 0.5) |> 
                     dplyr::mutate(dplyr::across(estimate:ci_u, ~round(.x, digits = 2)))",
                    "```"
  ),
  envir = environment(),
  quiet  = TRUE
  )
})

cat(unlist(res), sep = "\n")
```

### 4.2.1 Intrinsic motivation score

### 4.2.2 Integrated regulation score

### 4.2.3 Identified regulation score

### 4.2.4 Introjected regulation score

### 4.2.5 External regulation score

### 4.2.6 Amotivation score

# 5 Change in the motivational profile for physical activity

## 5.1 Assess cluster tendency

### 5.1.1 Graphically inspect data using principal component analysis

```
targets::tar_read(p_pca)
```

### 5.1.2 Confirm cluser tendency using graphical method

```
targets::tar_read(cluster_tendency)
```

Red: high similarity (ie: low dissimilarity) | Blue: low similarity

### 5.1.3 Confirm cluser tendency using the Hopkins statistic

#### 5.1.3.1 Hopkins statistic for the dataset at month 0

```
h_vec <- vector("double", 100)
set.seed(123)
seeds <- round(sample(rnorm(10000, 1000, 500), 100), 0)
for (i in 1:100) {
  set.seed(seeds[i])
  h_vec[i] <- hopkins(DB_EMAPS_0_12_M0_scaled)
}
mean(h_vec)
```

```
## [1] 0.9053554
```

**Comment**: Data seem clustered (Hopkins statistic
>0.7).

#### 5.1.3.2 Hopkins statistic for the dataset at month 12

```
h_vec <- vector("double", 100)
set.seed(123)
seeds <- round(sample(rnorm(10000, 1000, 500), 100), 0)
for (i in 1:100) {
  set.seed(seeds[i])
  h_vec[i] <- hopkins(DB_EMAPS_0_12_M12_scaled)
  
}
mean(h_vec)
```

```
## [1] 0.8935322
```

**Comment**: Data seem clustered (Hopkins statistic
>0.7).

## 5.2 Check outliers related to a multivariate analysis

### 5.2.1 Month 0

```
check_outliers(DB_EMAPS_0_12_M0_scaled, method = "mcd")
```

```
## 18 outliers detected: cases 4, 12, 13, 17, 20, 32, 41, 44, 53, 56, 60,
##   61, 64, 65, 67, 71, 75, 76.
## - Based on the following method and threshold: mcd (22.458).
## - For variables: INTRINSIC, INTEGRATED, IDENTIFIED, INTROJECTED,
##   EXTERNAL, AMOTIVATION.
```

### 5.2.2 Month 12

```
check_outliers(DB_EMAPS_0_12_M12_scaled[, c(names(DB_EMAPS_0_12_M12_scaled)[1:5])], method = "mcd")
```

```
## 20 outliers detected: cases 5, 13, 15, 16, 17, 19, 20, 21, 23, 27, 30,
##   31, 40, 48, 50, 55, 58, 61, 70, 74.
## - Based on the following method and threshold: mcd (20.515).
## - For variables: INTRINSIC, INTEGRATED, IDENTIFIED, INTROJECTED,
##   EXTERNAL.
```

**Comment**: We removed AMOTIVATION variable that caused
an error due to an IQR of 0.

## 5.3 Determine the optimal number of clusters related to a K-Medoid approach using the silhouette method

```
targets::tar_read(optim_n_clusters)
```

**Comment**: The optimal number of clusters related to
the K-Medoid approach is 2 at both 0 and 12 months post-program
(according to the silhouette method).

## 5.4 Visualize the final clusters from K-Medoid approach

```
targets::tar_read(final_clusters_emaps_viz)
```

## 5.5 Visualize the descriptive statistics of the EMAPS scores related to the motivational profiles

```
targets::tar_read(p_emaps_clust)
```

AU = Autonomous; IR = Introjected regulation.

```
targets::tar_read(table_stats_clusters_emaps)
```

## 5.6 Compare the motivational profiles

### 5.6.1 Comparison at Month 0

#### 5.6.1.1 Global analysis

```
targets::tar_read(nonpartest_global_month0)
```

```
## $results
##                                                   Test Statistic   df1      df2
## ANOVA type test p-value                                   39.282 4.316 310.9785
## McKeon approx. for the Lawley Hotelling Test                  NA    NA       NA
## Muller approx. for the Bartlett-Nanda-Pillai Test             NA    NA       NA
## Wilks Lambda                                                  NA    NA       NA
##                                                   P-value
## ANOVA type test p-value                                 0
## McKeon approx. for the Lawley Hotelling Test           NA
## Muller approx. for the Bartlett-Nanda-Pillai Test      NA
## Wilks Lambda                                           NA
##                                                   Permutation Test p-value
## ANOVA type test p-value                                                  0
## McKeon approx. for the Lawley Hotelling Test                            NA
## Muller approx. for the Bartlett-Nanda-Pillai Test                       NA
## Wilks Lambda                                                            NA
## 
## $twogroupreleffects
##                      INTRINSIC INTEGRATED IDENTIFIED INTROJECTED EXTERNAL
## High AU-Mod IR         0.11257    0.08062    0.09233     0.12713  0.52522
## Very High AU-High IR   0.88743    0.91938    0.90767     0.87287  0.47478
##                      AMOTIVATION
## High AU-Mod IR           0.52983
## Very High AU-High IR     0.47017
```

#### 5.6.1.2 Localisation of the difference(s)

```
ssnonpartest(INTRINSIC | INTEGRATED | IDENTIFIED | INTROJECTED | EXTERNAL | AMOTIVATION ~ cluster, data = tar_read(DB_EMAPS_0_12_clust) |> filter(MONTH == "0"), test = c(1, 0, 0, 0), alpha = 0.05, factors.and.variables = TRUE)
```

```
## 
## The ANOVA type statistic will be used in the following test 
## The Global Hypothesis is significant, subset algorithm will continue 
## 
## ~Performing the Subset Algorithm based on Factor levels~
## The Hypothesis of equality between factor levels  High AU-Mod IR Very High AU-High IR is rejected 
## All appropriate subsets using factor levels have been checked using a closed multiple testing procedure, which controls the maximum overall type I error rate at alpha= 0.05 
## 
## ~Performing the Subset Algorithm based on Response Variables~ 
##  The Hypothesis of equality using response variables  INTRINSIC INTEGRATED IDENTIFIED INTROJECTED EXTERNAL AMOTIVATION is rejected 
## The Hypothesis of equality using response variables  INTEGRATED IDENTIFIED INTROJECTED EXTERNAL AMOTIVATION is rejected 
## The Hypothesis of equality using response variables  INTRINSIC IDENTIFIED INTROJECTED EXTERNAL AMOTIVATION is rejected 
## The Hypothesis of equality using response variables  INTRINSIC INTEGRATED INTROJECTED EXTERNAL AMOTIVATION is rejected 
## The Hypothesis of equality using response variables  INTRINSIC INTEGRATED IDENTIFIED EXTERNAL AMOTIVATION is rejected 
## The Hypothesis of equality using response variables  INTRINSIC INTEGRATED IDENTIFIED INTROJECTED AMOTIVATION is rejected 
## The Hypothesis of equality using response variables  INTRINSIC INTEGRATED IDENTIFIED INTROJECTED EXTERNAL is rejected 
## The Hypothesis of equality using response variables  IDENTIFIED INTROJECTED EXTERNAL AMOTIVATION is rejected  
## The Hypothesis of equality using response variables  INTEGRATED INTROJECTED EXTERNAL AMOTIVATION is rejected  
## The Hypothesis of equality using response variables  INTEGRATED IDENTIFIED EXTERNAL AMOTIVATION is rejected  
## The Hypothesis of equality using response variables  INTEGRATED IDENTIFIED INTROJECTED AMOTIVATION is rejected  
## The Hypothesis of equality using response variables  INTEGRATED IDENTIFIED INTROJECTED EXTERNAL is rejected  
## The Hypothesis of equality using response variables  INTRINSIC INTROJECTED EXTERNAL AMOTIVATION is rejected  
## The Hypothesis of equality using response variables  INTRINSIC IDENTIFIED EXTERNAL AMOTIVATION is rejected  
## The Hypothesis of equality using response variables  INTRINSIC IDENTIFIED INTROJECTED AMOTIVATION is rejected  
## The Hypothesis of equality using response variables  INTRINSIC IDENTIFIED INTROJECTED EXTERNAL is rejected  
## The Hypothesis of equality using response variables  INTRINSIC INTEGRATED EXTERNAL AMOTIVATION is rejected  
## The Hypothesis of equality using response variables  INTRINSIC INTEGRATED INTROJECTED AMOTIVATION is rejected  
## The Hypothesis of equality using response variables  INTRINSIC INTEGRATED INTROJECTED EXTERNAL is rejected  
## The Hypothesis of equality using response variables  INTRINSIC INTEGRATED IDENTIFIED AMOTIVATION is rejected  
## The Hypothesis of equality using response variables  INTRINSIC INTEGRATED IDENTIFIED EXTERNAL is rejected  
## The Hypothesis of equality using response variables  INTRINSIC INTEGRATED IDENTIFIED INTROJECTED is rejected  
## The Hypothesis of equality using response variables  INTROJECTED EXTERNAL AMOTIVATION is rejected 
## The Hypothesis of equality using response variables  IDENTIFIED EXTERNAL AMOTIVATION is rejected 
## The Hypothesis of equality using response variables  IDENTIFIED INTROJECTED AMOTIVATION is rejected 
## The Hypothesis of equality using response variables  IDENTIFIED INTROJECTED EXTERNAL is rejected 
## The Hypothesis of equality using response variables  INTEGRATED EXTERNAL AMOTIVATION is rejected 
## The Hypothesis of equality using response variables  INTEGRATED INTROJECTED AMOTIVATION is rejected 
## The Hypothesis of equality using response variables  INTEGRATED INTROJECTED EXTERNAL is rejected 
## The Hypothesis of equality using response variables  INTEGRATED IDENTIFIED AMOTIVATION is rejected 
## The Hypothesis of equality using response variables  INTEGRATED IDENTIFIED EXTERNAL is rejected 
## The Hypothesis of equality using response variables  INTEGRATED IDENTIFIED INTROJECTED is rejected 
## The Hypothesis of equality using response variables  INTRINSIC EXTERNAL AMOTIVATION is rejected 
## The Hypothesis of equality using response variables  INTRINSIC INTROJECTED AMOTIVATION is rejected 
## The Hypothesis of equality using response variables  INTRINSIC INTROJECTED EXTERNAL is rejected 
## The Hypothesis of equality using response variables  INTRINSIC IDENTIFIED AMOTIVATION is rejected 
## The Hypothesis of equality using response variables  INTRINSIC IDENTIFIED EXTERNAL is rejected 
## The Hypothesis of equality using response variables  INTRINSIC IDENTIFIED INTROJECTED is rejected 
## The Hypothesis of equality using response variables  INTRINSIC INTEGRATED AMOTIVATION is rejected 
## The Hypothesis of equality using response variables  INTRINSIC INTEGRATED EXTERNAL is rejected 
## The Hypothesis of equality using response variables  INTRINSIC INTEGRATED INTROJECTED is rejected 
## The Hypothesis of equality using response variables  INTRINSIC INTEGRATED IDENTIFIED is rejected 
## The Hypothesis of equality using response variables  INTROJECTED AMOTIVATION is rejected 
## The Hypothesis of equality using response variables  INTROJECTED EXTERNAL is rejected 
## The Hypothesis of equality using response variables  IDENTIFIED AMOTIVATION is rejected 
## The Hypothesis of equality using response variables  IDENTIFIED EXTERNAL is rejected 
## The Hypothesis of equality using response variables  IDENTIFIED INTROJECTED is rejected 
## The Hypothesis of equality using response variables  INTEGRATED AMOTIVATION is rejected 
## The Hypothesis of equality using response variables  INTEGRATED EXTERNAL is rejected 
## The Hypothesis of equality using response variables  INTEGRATED INTROJECTED is rejected 
## The Hypothesis of equality using response variables  INTEGRATED IDENTIFIED is rejected 
## The Hypothesis of equality using response variables  INTRINSIC AMOTIVATION is rejected 
## The Hypothesis of equality using response variables  INTRINSIC EXTERNAL is rejected 
## The Hypothesis of equality using response variables  INTRINSIC INTROJECTED is rejected 
## The Hypothesis of equality using response variables  INTRINSIC IDENTIFIED is rejected 
## The Hypothesis of equality using response variables  INTRINSIC INTEGRATED is rejected 
## The Hypothesis of equality using response variables  INTROJECTED is rejected 
## The Hypothesis of equality using response variables  IDENTIFIED is rejected 
## The Hypothesis of equality using response variables  INTEGRATED is rejected 
## The Hypothesis of equality using response variables  INTRINSIC is rejected 
## All appropriate subsets using response variables have been checked using a multiple testing procedure, which controls the maximum overall type I error rate at alpha= 0.05
```

### 5.6.2 Comparison at Month 12

#### 5.6.2.1 Global analysis

```
targets::tar_read(nonpartest_global_month12)
```

```
## $results
##                                                   Test Statistic   df1      df2
## ANOVA type test p-value                                   52.305 4.353 298.6204
## McKeon approx. for the Lawley Hotelling Test              32.769 6.000  69.0000
## Muller approx. for the Bartlett-Nanda-Pillai Test         32.337 6.074  68.9316
## Wilks Lambda                                              32.769 6.000  69.0000
##                                                   P-value
## ANOVA type test p-value                                 0
## McKeon approx. for the Lawley Hotelling Test            0
## Muller approx. for the Bartlett-Nanda-Pillai Test       0
## Wilks Lambda                                            0
##                                                   Permutation Test p-value
## ANOVA type test p-value                                                  0
## McKeon approx. for the Lawley Hotelling Test                             0
## Muller approx. for the Bartlett-Nanda-Pillai Test                        0
## Wilks Lambda                                                             0
## 
## $twogroupreleffects
##                      INTRINSIC INTEGRATED IDENTIFIED INTROJECTED EXTERNAL
## High AU-Mod IR         0.08445    0.06436     0.0997     0.07776  0.59561
## Very High AU-High IR   0.91555    0.93564     0.9003     0.92224  0.40439
##                      AMOTIVATION
## High AU-Mod IR           0.66666
## Very High AU-High IR     0.33334
```

#### 5.6.2.2 Localisation of the difference(s)

```
ssnonpartest(INTRINSIC | INTEGRATED | IDENTIFIED | INTROJECTED | EXTERNAL | AMOTIVATION ~ cluster, data = tar_read(DB_EMAPS_0_12_clust) |> filter(MONTH == "12"), test = c(1, 0, 0, 0), alpha = 0.05, factors.and.variables = TRUE)
```

```
## 
## The ANOVA type statistic will be used in the following test 
## The Global Hypothesis is significant, subset algorithm will continue 
## 
## ~Performing the Subset Algorithm based on Factor levels~
## The Hypothesis of equality between factor levels  High AU-Mod IR Very High AU-High IR is rejected 
## All appropriate subsets using factor levels have been checked using a closed multiple testing procedure, which controls the maximum overall type I error rate at alpha= 0.05 
## 
## ~Performing the Subset Algorithm based on Response Variables~ 
##  The Hypothesis of equality using response variables  INTRINSIC INTEGRATED IDENTIFIED INTROJECTED EXTERNAL AMOTIVATION is rejected 
## The Hypothesis of equality using response variables  INTEGRATED IDENTIFIED INTROJECTED EXTERNAL AMOTIVATION is rejected 
## The Hypothesis of equality using response variables  INTRINSIC IDENTIFIED INTROJECTED EXTERNAL AMOTIVATION is rejected 
## The Hypothesis of equality using response variables  INTRINSIC INTEGRATED INTROJECTED EXTERNAL AMOTIVATION is rejected 
## The Hypothesis of equality using response variables  INTRINSIC INTEGRATED IDENTIFIED EXTERNAL AMOTIVATION is rejected 
## The Hypothesis of equality using response variables  INTRINSIC INTEGRATED IDENTIFIED INTROJECTED AMOTIVATION is rejected 
## The Hypothesis of equality using response variables  INTRINSIC INTEGRATED IDENTIFIED INTROJECTED EXTERNAL is rejected 
## The Hypothesis of equality using response variables  IDENTIFIED INTROJECTED EXTERNAL AMOTIVATION is rejected  
## The Hypothesis of equality using response variables  INTEGRATED INTROJECTED EXTERNAL AMOTIVATION is rejected  
## The Hypothesis of equality using response variables  INTEGRATED IDENTIFIED EXTERNAL AMOTIVATION is rejected  
## The Hypothesis of equality using response variables  INTEGRATED IDENTIFIED INTROJECTED AMOTIVATION is rejected  
## The Hypothesis of equality using response variables  INTEGRATED IDENTIFIED INTROJECTED EXTERNAL is rejected  
## The Hypothesis of equality using response variables  INTRINSIC INTROJECTED EXTERNAL AMOTIVATION is rejected  
## The Hypothesis of equality using response variables  INTRINSIC IDENTIFIED EXTERNAL AMOTIVATION is rejected  
## The Hypothesis of equality using response variables  INTRINSIC IDENTIFIED INTROJECTED AMOTIVATION is rejected  
## The Hypothesis of equality using response variables  INTRINSIC IDENTIFIED INTROJECTED EXTERNAL is rejected  
## The Hypothesis of equality using response variables  INTRINSIC INTEGRATED EXTERNAL AMOTIVATION is rejected  
## The Hypothesis of equality using response variables  INTRINSIC INTEGRATED INTROJECTED AMOTIVATION is rejected  
## The Hypothesis of equality using response variables  INTRINSIC INTEGRATED INTROJECTED EXTERNAL is rejected  
## The Hypothesis of equality using response variables  INTRINSIC INTEGRATED IDENTIFIED AMOTIVATION is rejected  
## The Hypothesis of equality using response variables  INTRINSIC INTEGRATED IDENTIFIED EXTERNAL is rejected  
## The Hypothesis of equality using response variables  INTRINSIC INTEGRATED IDENTIFIED INTROJECTED is rejected  
## The Hypothesis of equality using response variables  INTROJECTED EXTERNAL AMOTIVATION is rejected 
## The Hypothesis of equality using response variables  IDENTIFIED EXTERNAL AMOTIVATION is rejected 
## The Hypothesis of equality using response variables  IDENTIFIED INTROJECTED AMOTIVATION is rejected 
## The Hypothesis of equality using response variables  IDENTIFIED INTROJECTED EXTERNAL is rejected 
## The Hypothesis of equality using response variables  INTEGRATED EXTERNAL AMOTIVATION is rejected 
## The Hypothesis of equality using response variables  INTEGRATED INTROJECTED AMOTIVATION is rejected 
## The Hypothesis of equality using response variables  INTEGRATED INTROJECTED EXTERNAL is rejected 
## The Hypothesis of equality using response variables  INTEGRATED IDENTIFIED AMOTIVATION is rejected 
## The Hypothesis of equality using response variables  INTEGRATED IDENTIFIED EXTERNAL is rejected 
## The Hypothesis of equality using response variables  INTEGRATED IDENTIFIED INTROJECTED is rejected 
## The Hypothesis of equality using response variables  INTRINSIC EXTERNAL AMOTIVATION is rejected 
## The Hypothesis of equality using response variables  INTRINSIC INTROJECTED AMOTIVATION is rejected 
## The Hypothesis of equality using response variables  INTRINSIC INTROJECTED EXTERNAL is rejected 
## The Hypothesis of equality using response variables  INTRINSIC IDENTIFIED AMOTIVATION is rejected 
## The Hypothesis of equality using response variables  INTRINSIC IDENTIFIED EXTERNAL is rejected 
## The Hypothesis of equality using response variables  INTRINSIC IDENTIFIED INTROJECTED is rejected 
## The Hypothesis of equality using response variables  INTRINSIC INTEGRATED AMOTIVATION is rejected 
## The Hypothesis of equality using response variables  INTRINSIC INTEGRATED EXTERNAL is rejected 
## The Hypothesis of equality using response variables  INTRINSIC INTEGRATED INTROJECTED is rejected 
## The Hypothesis of equality using response variables  INTRINSIC INTEGRATED IDENTIFIED is rejected 
## The Hypothesis of equality using response variables  INTROJECTED AMOTIVATION is rejected 
## The Hypothesis of equality using response variables  INTROJECTED EXTERNAL is rejected 
## The Hypothesis of equality using response variables  IDENTIFIED AMOTIVATION is rejected 
## The Hypothesis of equality using response variables  IDENTIFIED EXTERNAL is rejected 
## The Hypothesis of equality using response variables  IDENTIFIED INTROJECTED is rejected 
## The Hypothesis of equality using response variables  INTEGRATED AMOTIVATION is rejected 
## The Hypothesis of equality using response variables  INTEGRATED EXTERNAL is rejected 
## The Hypothesis of equality using response variables  INTEGRATED INTROJECTED is rejected 
## The Hypothesis of equality using response variables  INTEGRATED IDENTIFIED is rejected 
## The Hypothesis of equality using response variables  INTRINSIC AMOTIVATION is rejected 
## The Hypothesis of equality using response variables  INTRINSIC EXTERNAL is rejected 
## The Hypothesis of equality using response variables  INTRINSIC INTROJECTED is rejected 
## The Hypothesis of equality using response variables  INTRINSIC IDENTIFIED is rejected 
## The Hypothesis of equality using response variables  INTRINSIC INTEGRATED is rejected 
## The Hypothesis of equality using response variables  INTROJECTED is rejected 
## The Hypothesis of equality using response variables  IDENTIFIED is rejected 
## The Hypothesis of equality using response variables  INTEGRATED is rejected 
## The Hypothesis of equality using response variables  INTRINSIC is rejected 
## All appropriate subsets using response variables have been checked using a multiple testing procedure, which controls the maximum overall type I error rate at alpha= 0.05
```

## 5.7 Characterize the change in the motivational profile

### 5.7.1 Visualize the change in the motivational profile

```
targets::tar_read(p_change_emaps_profile_alluvial)
```

### 5.7.2 Compute the proportions of patients per profile transition scenario

```
tar_read(prop_trans_prof_motiv)
```

# 6 Barriers to physical activity at 12 months

```
targets::tar_read(p_BARRIERS)
```

Barriers to physical activity (N = 77). Answers have been translated
from French to English for the reader�s understanding of the figure. The
most frequently evocated barriers have been highlighted with darker
colours.

# 7 Predict 6WT distance trajectory using latent class mixed modelling

## 7.1 Build the models

```
# Load the model summarises
tar_load(model_6MWT_n1)
tar_load(model_6MWT_n2)
tar_load(model_6MWT_n3)
tar_load(model_6MWT_n4)
tar_load(model_6MWT_n5)
```

### 7.1.1 1-class model

```
summary(model_6MWT_n1)
```

```
## Heterogenous linear mixed model 
##      fitted by maximum likelihood method 
##  
## hlme(fixed = DIST_6MWT ~ MONTH, random = ~1, subject = "patient", 
##     ng = 1, data = DB_PRED_6MWT_0_12, var.time = "MONTH")
##  
## Statistical Model: 
##      Dataset: DB_PRED_6MWT_0_12 
##      Number of subjects: 75 
##      Number of observations: 150 
##      Number of latent classes: 1 
##      Number of parameters: 4  
##  
## Iteration process: 
##      Convergence criteria satisfied 
##      Number of iterations:  19 
##      Convergence criteria: parameters= 4.7e-10 
##                          : likelihood= 2.6e-12 
##                          : second derivatives= 1.1e-18 
##  
## Goodness-of-fit statistics: 
##      maximum log-likelihood: -807.98  
##      AIC: 1623.96  
##      BIC: 1633.23  
##  
##  
## Maximum Likelihood Estimates: 
##  
## Fixed effects in the longitudinal model:
## 
##                 coef      Se     Wald p-value
## intercept  605.44000 8.81756   68.663 0.00000
## MONTH        1.04667 0.36334    2.881 0.00397
## 
## 
## Variance-covariance matrix of the random-effects:
##           intercept
## intercept  5118.307
## 
##                                coef      Se
## Residual standard error:   26.70012 2.18060
```

### 7.1.2 2-class model

```
summary(model_6MWT_n2)
```

```
## Heterogenous linear mixed model 
##      fitted by maximum likelihood method 
##  
## hlme(fixed = DIST_6MWT ~ MONTH, mixture = ~MONTH, random = ~1, 
##     subject = "patient", ng = 2, data = DB_PRED_6MWT_0_12, var.time = "MONTH")
##  
## Statistical Model: 
##      Dataset: DB_PRED_6MWT_0_12 
##      Number of subjects: 75 
##      Number of observations: 150 
##      Number of latent classes: 2 
##      Number of parameters: 7  
##  
## Iteration process: 
##      Convergence criteria satisfied 
##      Number of iterations:  1 
##      Convergence criteria: parameters= 1e-10 
##                          : likelihood= 3.4e-13 
##                          : second derivatives= 3e-14 
##  
## Goodness-of-fit statistics: 
##      maximum log-likelihood: -804.75  
##      AIC: 1623.5  
##      BIC: 1639.72  
##  
##  
## Maximum Likelihood Estimates: 
##  
## Fixed effects in the class-membership model:
## (the class of reference is the last class) 
## 
##                        coef       Se     Wald p-value
## intercept class1    0.92959  0.78224    1.188 0.23469
## 
## Fixed effects in the longitudinal model:
## 
##                        coef       Se     Wald p-value
## intercept class1  618.45200 10.92629   56.602 0.00000
## intercept class2  572.47456 24.12087   23.734 0.00000
## MONTH class1        2.19493  0.64589    3.398 0.00068
## MONTH class2       -1.86242  1.09135   -1.707 0.08791
## 
## 
## Variance-covariance matrix of the random-effects:
##           intercept
## intercept  4235.124
## 
##                                coef       Se
## Residual standard error:   21.73450  2.55354
```

### 7.1.3 3-class model

```
summary(model_6MWT_n3)
```

```
## Heterogenous linear mixed model 
##      fitted by maximum likelihood method 
##  
## hlme(fixed = DIST_6MWT ~ MONTH, mixture = ~MONTH, random = ~1, 
##     subject = "patient", ng = 3, data = DB_PRED_6MWT_0_12, var.time = "MONTH")
##  
## Statistical Model: 
##      Dataset: DB_PRED_6MWT_0_12 
##      Number of subjects: 75 
##      Number of observations: 150 
##      Number of latent classes: 3 
##      Number of parameters: 10  
##  
## Iteration process: 
##      Convergence criteria satisfied 
##      Number of iterations:  1 
##      Convergence criteria: parameters= 9.8e-11 
##                          : likelihood= 2.3e-13 
##                          : second derivatives= 9.1e-15 
##  
## Goodness-of-fit statistics: 
##      maximum log-likelihood: -799.71  
##      AIC: 1619.42  
##      BIC: 1642.6  
##  
##  
## Maximum Likelihood Estimates: 
##  
## Fixed effects in the class-membership model:
## (the class of reference is the last class) 
## 
##                        coef       Se     Wald p-value
## intercept class1    2.93787  0.74700    3.933 0.00008
## intercept class2    2.81747  0.75951    3.710 0.00021
## 
## Fixed effects in the longitudinal model:
## 
##                        coef       Se     Wald p-value
## intercept class1  588.73829 12.39353   47.504 0.00000
## intercept class2  617.86859 12.67690   48.740 0.00000
## intercept class3  712.71000 52.53194   13.567 0.00000
## MONTH class1       -0.70141  0.44815   -1.565 0.11756
## MONTH class2        3.56567  0.51972    6.861 0.00000
## MONTH class3       -8.11139  1.32606   -6.117 0.00000
## 
## 
## Variance-covariance matrix of the random-effects:
##           intercept
## intercept   4559.21
## 
##                                coef       Se
## Residual standard error:   15.02270  1.81554
```

### 7.1.4 4-class model

```
summary(model_6MWT_n4)
```

```
## Heterogenous linear mixed model 
##      fitted by maximum likelihood method 
##  
## hlme(fixed = DIST_6MWT ~ MONTH, mixture = ~MONTH, random = ~1, 
##     subject = "patient", ng = 4, data = DB_PRED_6MWT_0_12, var.time = "MONTH")
##  
## Statistical Model: 
##      Dataset: DB_PRED_6MWT_0_12 
##      Number of subjects: 75 
##      Number of observations: 150 
##      Number of latent classes: 4 
##      Number of parameters: 13  
##  
## Iteration process: 
##      Convergence criteria satisfied 
##      Number of iterations:  1 
##      Convergence criteria: parameters= 1e-10 
##                          : likelihood= 1.1e-13 
##                          : second derivatives= 2e-14 
##  
## Goodness-of-fit statistics: 
##      maximum log-likelihood: -795.67  
##      AIC: 1617.34  
##      BIC: 1647.47  
##  
##  
## Maximum Likelihood Estimates: 
##  
## Fixed effects in the class-membership model:
## (the class of reference is the last class) 
## 
##                        coef       Se     Wald p-value
## intercept class1    1.67944  0.85377    1.967 0.04917
## intercept class2    1.32031  0.85122    1.551 0.12088
## intercept class3    3.27207  0.73008    4.482 0.00001
## 
## Fixed effects in the longitudinal model:
## 
##                        coef       Se     Wald p-value
## intercept class1  517.47416 21.95123   23.574 0.00000
## intercept class2  736.76149 24.08361   30.592 0.00000
## intercept class3  600.46864  7.75010   77.479 0.00000
## intercept class4  716.48954 32.14137   22.292 0.00000
## MONTH class1       -1.94655  0.81868   -2.378 0.01742
## MONTH class2        2.00029  1.02097    1.959 0.05009
## MONTH class3        1.86415  0.41295    4.514 0.00001
## MONTH class4       -8.02674  1.85553   -4.326 0.00002
## 
## 
## Variance-covariance matrix of the random-effects:
##           intercept
## intercept  1602.019
## 
##                                coef       Se
## Residual standard error:   20.34771  1.89292
```

### 7.1.5 5-class model

```
summary(model_6MWT_n5)
```

```
## Heterogenous linear mixed model 
##      fitted by maximum likelihood method 
##  
## hlme(fixed = DIST_6MWT ~ MONTH, mixture = ~MONTH, random = ~1, 
##     subject = "patient", ng = 5, data = DB_PRED_6MWT_0_12, var.time = "MONTH")
##  
## Statistical Model: 
##      Dataset: DB_PRED_6MWT_0_12 
##      Number of subjects: 75 
##      Number of observations: 150 
##      Number of latent classes: 5 
##      Number of parameters: 16  
##  
## Iteration process: 
##      Convergence criteria satisfied 
##      Number of iterations:  1 
##      Convergence criteria: parameters= 1.9e-05 
##                          : likelihood= 2.3e-08 
##                          : second derivatives= 1.1e-10 
##  
## Goodness-of-fit statistics: 
##      maximum log-likelihood: -793.24  
##      AIC: 1618.48  
##      BIC: 1655.56  
##  
##  
## Maximum Likelihood Estimates: 
##  
## Fixed effects in the class-membership model:
## (the class of reference is the last class) 
## 
##                        coef       Se     Wald p-value
## intercept class1    1.47921  0.64617    2.289 0.02207
## intercept class2    1.16064  0.72325    1.605 0.10855
## intercept class3   -1.34808  0.90409   -1.491 0.13594
## intercept class4   -0.18085  0.73602   -0.246 0.80590
## 
## Fixed effects in the longitudinal model:
## 
##                        coef       Se     Wald p-value
## intercept class1  596.44041 10.53783   56.600 0.00000
## intercept class2  603.49269 11.01307   54.798 0.00000
## intercept class3  716.99703 31.31971   22.893 0.00000
## intercept class4  748.60884 23.42899   31.952 0.00000
## intercept class5  502.70072 25.53577   19.686 0.00000
## MONTH class1        0.17891  0.59780    0.299 0.76473
## MONTH class2        3.90563  0.71218    5.484 0.00000
## MONTH class3       -8.16887  1.34904   -6.055 0.00000
## MONTH class4        1.53208  0.92494    1.656 0.09764
## MONTH class5       -2.28154  0.78589   -2.903 0.00369
## 
## 
## Variance-covariance matrix of the random-effects:
##           intercept
## intercept  1702.061
## 
##                                coef       Se
## Residual standard error:   15.68832  1.95686
```

## 7.2 Compare the models

### 7.2.1 Analyse the metrics of the models

```
tar_read(compa_latent_mixed_models_table_6MWT)
```

```
##                    AIC      BIC   entropy   %class1   %class2   %class3
## model_6MWT_n1 1623.961 1633.230 1.0000000 100.00000        NA        NA
## model_6MWT_n2 1623.495 1639.718 0.5212070  74.66667 25.333333        NA
## model_6MWT_n3 1619.422 1642.597 0.7691509  52.00000 45.333333  2.666667
## model_6MWT_n4 1617.344 1647.471 0.8647545  13.33333  9.333333 74.666667
## model_6MWT_n5 1618.482 1655.562 0.7620378  48.00000 30.666667  2.666667
##                %class4  %class5
## model_6MWT_n1       NA       NA
## model_6MWT_n2       NA       NA
## model_6MWT_n3       NA       NA
## model_6MWT_n4 2.666667       NA
## model_6MWT_n5 8.000000 10.66667
```

```
lcmm::summaryplot(
  model_6MWT_n1,
  model_6MWT_n2,
  model_6MWT_n3,
  model_6MWT_n4,
  model_6MWT_n5,
  which = c("AIC", "BIC", "entropy")
)
```

### 7.2.2 Posterior classification of the models

#### 7.2.2.1 2-class model

```
lcmm::postprob(model_6MWT_n2)
```

```
##  
## Posterior classification: 
##   class1 class2
## N  56.00  19.00
## %  74.67  25.33
##  
## Posterior classification table: 
##      --> mean of posterior probabilities in each class 
##         prob1  prob2
## class1 0.8855 0.1145
## class2 0.2202 0.7798
##  
## Posterior probabilities above a threshold (%): 
##          class1 class2
## prob>0.7  92.86  68.42
## prob>0.8  76.79  42.11
## prob>0.9  60.71  26.32
##
```

#### 7.2.2.2 3-class model

```
lcmm::postprob(model_6MWT_n3)
```

```
##  
## Posterior classification: 
##   class1 class2 class3
## N     39  34.00   2.00
## %     52  45.33   2.67
##  
## Posterior classification table: 
##      --> mean of posterior probabilities in each class 
##         prob1  prob2  prob3
## class1 0.8895 0.1089 0.0016
## class2 0.1166 0.8834 0.0000
## class3 0.0064 0.0000 0.9936
##  
## Posterior probabilities above a threshold (%): 
##          class1 class2 class3
## prob>0.7  84.62  85.29    100
## prob>0.8  76.92  79.41    100
## prob>0.9  66.67  61.76    100
##
```

#### 7.2.2.3 4-class model

```
lcmm::postprob(model_6MWT_n4)
```

```
##  
## Posterior classification: 
##   class1 class2 class3 class4
## N  10.00   7.00  56.00   2.00
## %  13.33   9.33  74.67   2.67
##  
## Posterior classification table: 
##      --> mean of posterior probabilities in each class 
##         prob1  prob2  prob3  prob4
## class1 0.8703 0.0000 0.1296 0.0001
## class2 0.0000 0.9190 0.0776 0.0034
## class3 0.0415 0.0226 0.9352 0.0007
## class4 0.0002 0.0009 0.0033 0.9957
##  
## Posterior probabilities above a threshold (%): 
##          class1 class2 class3 class4
## prob>0.7     80  85.71  94.64    100
## prob>0.8     80  85.71  87.50    100
## prob>0.9     50  71.43  82.14    100
##
```

#### 7.2.2.4 5-class model

```
lcmm::postprob(model_6MWT_n5)
```

```
##  
## Posterior classification: 
##   class1 class2 class3 class4 class5
## N     36  23.00   2.00      6   8.00
## %     48  30.67   2.67      8  10.67
##  
## Posterior classification table: 
##      --> mean of posterior probabilities in each class 
##         prob1  prob2  prob3  prob4  prob5
## class1 0.8150 0.1445 0.0002 0.0061 0.0343
## class2 0.1355 0.8385 0.0000 0.0260 0.0000
## class3 0.0006 0.0000 0.9994 0.0000 0.0000
## class4 0.0152 0.0414 0.0013 0.9422 0.0000
## class5 0.1847 0.0007 0.0001 0.0000 0.8145
##  
## Posterior probabilities above a threshold (%): 
##          class1 class2 class3 class4 class5
## prob>0.7  75.00  78.26    100 100.00   62.5
## prob>0.8  61.11  65.22    100 100.00   62.5
## prob>0.9  38.89  47.83    100  83.33   62.5
##
```

**Comment**: Based on the metrics shown above, the
4-class model could seem to be the best model but the 3-class model also
showed good metrics and had less classes containing very few
participants compared to the 4-class model. Thus the 3-class model was
chosen.

## 7.3 Permut the classes of the chosen model

```
tar_load(model_6MWT_n3_permut)
summary(model_6MWT_n3_permut)
```

```
## Heterogenous linear mixed model 
##      fitted by maximum likelihood method 
##  
## hlme(fixed = DIST_6MWT ~ MONTH, mixture = ~MONTH, random = ~1, 
##     subject = "patient", ng = 3, data = DB_PRED_6MWT_0_12, var.time = "MONTH")
##  
## Statistical Model: 
##      Dataset: DB_PRED_6MWT_0_12 
##      Number of subjects: 75 
##      Number of observations: 150 
##      Number of latent classes: 3 
##      Number of parameters: 10  
##  
## Iteration process: 
##      Convergence criteria satisfied 
##      Number of iterations:  1 
##      Convergence criteria: parameters= 2.7e-11 
##                          : likelihood= 3.4e-13 
##                          : second derivatives= 6.8e-16 
##  
## Goodness-of-fit statistics: 
##      maximum log-likelihood: -799.71  
##      AIC: 1619.42  
##      BIC: 1642.6  
##  
##  
## Maximum Likelihood Estimates: 
##  
## Fixed effects in the class-membership model:
## (the class of reference is the last class) 
## 
##                        coef       Se     Wald p-value
## intercept class1   -2.81747  0.75945   -3.710 0.00021
## intercept class2    0.12040  0.40895    0.294 0.76843
## 
## Fixed effects in the longitudinal model:
## 
##                        coef       Se     Wald p-value
## intercept class1  712.71000 52.53197   13.567 0.00000
## intercept class2  588.73829 12.39370   47.503 0.00000
## intercept class3  617.86859 12.67692   48.740 0.00000
## MONTH class1       -8.11139  1.32607   -6.117 0.00000
## MONTH class2       -0.70141  0.44820   -1.565 0.11760
## MONTH class3        3.56567  0.51977    6.860 0.00000
## 
## 
## Variance-covariance matrix of the random-effects:
##           intercept
## intercept   4559.21
## 
##                                coef       Se
## Residual standard error:   15.02270  1.81553
```

```
lcmm::postprob(model_6MWT_n3_permut)
```

```
##  
## Posterior classification: 
##   class1 class2 class3
## N   2.00     39  34.00
## %   2.67     52  45.33
##  
## Posterior classification table: 
##      --> mean of posterior probabilities in each class 
##         prob1  prob2  prob3
## class1 0.9936 0.0064 0.0000
## class2 0.0016 0.8895 0.1089
## class3 0.0000 0.1166 0.8834
##  
## Posterior probabilities above a threshold (%): 
##          class1 class2 class3
## prob>0.7    100  84.62  85.29
## prob>0.8    100  76.92  79.41
## prob>0.9    100  66.67  61.76
##
```

## 7.4 Assess the chosen model

```
plot(model_6MWT_n3_permut)
```

## 7.5 Visualize the fixed effetcs of the model

```
tar_read(plot_preds_6MWT)
```

## 7.6 Analyse the predictors of the latent classes of the model

```
tar_load(predictors_6MWT_classes)
summary(predictors_6MWT_classes)
```

```
## Secondary multinomial model for external class predictor 
##      fitted by maximum likelihood method 
##  
## externVar(model = model_6MWT_n3_permut, subject = "patient", 
##     classmb = ~DIST_6MWT_M0 + MET_MIN_WK_M0 + MOTIVATION_CLUSTER_M0 + 
##         meteo_defavorable + manque_temps, data = DB_PRED_6MWT_0_12, 
##     method = "twoStageJoint")
##  
## Statistical Model: 
##      Dataset: DB_PRED_6MWT_0_12 
##      Number of subjects: 74 
##      Number of latent classes: 3 
##      Number of parameters: 12  
##  
## Iteration process: 
##      Convergence criteria satisfied 
##      Number of iterations:  65 
##      Convergence criteria: parameters= 9.9e-05 
##                          : likelihood= 2.6e-06 
##                          : second derivatives= 4.3e-05 
##  
## Goodness-of-fit statistics: 
##      maximum log-likelihood: -780.17  
##      AIC: 1584.34  
##      BIC: 1611.99  
##  
##  
##  
## Maximum Likelihood Estimates: 
##  
## Fixed effects in the class-membership model:
## (the class of reference is the last class) 
## 
##                                                       coef     Se**   Wald
## intercept class1                                 -17.20337 62.36430 -0.276
## intercept class2                                   7.60341  7.74625  0.982
## DIST_6MWT_M0 class1                                0.01139  0.01428  0.798
## DIST_6MWT_M0 class2                               -0.01481  0.01463 -1.012
## MET_MIN_WK_M0 class1                              -0.00024  0.00101 -0.235
## MET_MIN_WK_M0 class2                               0.00012  0.00013  0.909
## MOTIVATION_CLUSTER_M0Very High AU-High IR class1   9.12534 61.89733  0.147
## MOTIVATION_CLUSTER_M0Very High AU-High IR class2   0.40474  0.73971  0.547
## meteo_defavorable1 class1                         -7.54157 88.84566 -0.085
## meteo_defavorable1 class2                          0.46055  0.91156  0.505
## manque_temps1 class1                              -7.17773 89.33639 -0.080
## manque_temps1 class2                               1.25477  1.31232  0.956
##                                                  p-value
## intercept class1                                 0.78266
## intercept class2                                 0.32632
## DIST_6MWT_M0 class1                              0.42501
## DIST_6MWT_M0 class2                              0.31145
## MET_MIN_WK_M0 class1                             0.81434
## MET_MIN_WK_M0 class2                             0.36331
## MOTIVATION_CLUSTER_M0Very High AU-High IR class1 0.88279
## MOTIVATION_CLUSTER_M0Very High AU-High IR class2 0.58427
## meteo_defavorable1 class1                        0.93235
## meteo_defavorable1 class2                        0.61339
## manque_temps1 class1                             0.93596
## manque_temps1 class2                             0.33900
## 
##  ** total variance estimated through the Hessian of the joint likelihood 
##
```

# 8 Predict IPAQ-SF MET-min/week trajectory using latent class mixed modelling

## 8.1 Build the models

```
# Load the model summarises
tar_load(model_IPAQ_n1)
tar_load(model_IPAQ_n2)
tar_load(model_IPAQ_n3)
tar_load(model_IPAQ_n4)
tar_load(model_IPAQ_n5)
```

### 8.1.1 1-class model

```
summary(model_IPAQ_n1)
```

```
## Heterogenous linear mixed model 
##      fitted by maximum likelihood method 
##  
## hlme(fixed = MET_MIN_WK ~ MONTH, random = ~1, subject = "patient", 
##     ng = 1, data = DB_PRED_IPAQ_6_12, var.time = "MONTH")
##  
## Statistical Model: 
##      Dataset: DB_PRED_IPAQ_6_12 
##      Number of subjects: 77 
##      Number of observations: 154 
##      Number of latent classes: 1 
##      Number of parameters: 4  
##  
## Iteration process: 
##      Convergence criteria satisfied 
##      Number of iterations:  34 
##      Convergence criteria: parameters= 1.5e-10 
##                          : likelihood= 4.5e-13 
##                          : second derivatives= 3.5e-17 
##  
## Goodness-of-fit statistics: 
##      maximum log-likelihood: -1488.88  
##      AIC: 2985.76  
##      BIC: 2995.14  
##  
##  
## Maximum Likelihood Estimates: 
##  
## Fixed effects in the longitudinal model:
## 
##                    coef        Se        Wald p-value
## intercept    3807.59740 800.89659       4.754 0.00000
## MONTH          32.05195  76.00845       0.422 0.67325
## 
## 
## Variance-covariance matrix of the random-effects:
##           intercept
## intercept   9355385
## 
##                                   coef        Se
## Residual standard error:    2829.45253 228.05682
```

### 8.1.2 2-class model

```
summary(model_IPAQ_n2)
```

```
## Heterogenous linear mixed model 
##      fitted by maximum likelihood method 
##  
## hlme(fixed = MET_MIN_WK ~ MONTH, mixture = ~MONTH, random = ~1, 
##     subject = "patient", ng = 2, data = DB_PRED_IPAQ_6_12, var.time = "MONTH")
##  
## Statistical Model: 
##      Dataset: DB_PRED_IPAQ_6_12 
##      Number of subjects: 77 
##      Number of observations: 154 
##      Number of latent classes: 2 
##      Number of parameters: 7  
##  
## Iteration process: 
##      Convergence criteria satisfied 
##      Number of iterations:  1 
##      Convergence criteria: parameters= 2.3e-10 
##                          : likelihood= 9.1e-13 
##                          : second derivatives= 7.2e-15 
##  
## Goodness-of-fit statistics: 
##      maximum log-likelihood: -1452.14  
##      AIC: 2918.28  
##      BIC: 2934.69  
##  
##  
## Maximum Likelihood Estimates: 
##  
## Fixed effects in the class-membership model:
## (the class of reference is the last class) 
## 
##                           coef         Se        Wald p-value
## intercept class1       2.67999    0.46854       5.720 0.00000
## 
## Fixed effects in the longitudinal model:
## 
##                           coef         Se        Wald p-value
## intercept class1    3840.89686  652.94808       5.882 0.00000
## intercept class2    3321.92590 2508.72561       1.324 0.18545
## MONTH class1         -58.71239   67.13797      -0.875 0.38184
## MONTH class2        1355.84623  261.80607       5.179 0.00000
## 
## 
## Variance-covariance matrix of the random-effects:
##           intercept
## intercept   1481643
## 
##                                   coef         Se
## Residual standard error:    2417.23850  195.11440
```

### 8.1.3 3-class model

```
summary(model_IPAQ_n3)
```

```
## Heterogenous linear mixed model 
##      fitted by maximum likelihood method 
##  
## hlme(fixed = MET_MIN_WK ~ MONTH, mixture = ~MONTH, random = ~1, 
##     subject = "patient", ng = 3, data = DB_PRED_IPAQ_6_12, var.time = "MONTH")
##  
## Statistical Model: 
##      Dataset: DB_PRED_IPAQ_6_12 
##      Number of subjects: 77 
##      Number of observations: 154 
##      Number of latent classes: 3 
##      Number of parameters: 10  
##  
## Iteration process: 
##      Convergence criteria satisfied 
##      Number of iterations:  10 
##      Convergence criteria: parameters= 1e-09 
##                          : likelihood= 2.3e-13 
##                          : second derivatives= 1.3e-17 
##  
## Goodness-of-fit statistics: 
##      maximum log-likelihood: -1448.61  
##      AIC: 2917.21  
##      BIC: 2940.65  
##  
##  
## Maximum Likelihood Estimates: 
##  
## Fixed effects in the class-membership model:
## (the class of reference is the last class) 
## 
##                          coef         Se       Wald p-value
## intercept class1      2.48601    0.69032      3.601 0.00032
## intercept class2     -0.32362    0.82697     -0.391 0.69556
## 
## Fixed effects in the longitudinal model:
## 
##                          coef         Se       Wald p-value
## intercept class1   3175.94038  668.19712      4.753 0.00000
## intercept class2   2157.01113 2630.67708      0.820 0.41225
## intercept class3  12590.06427 3862.36130      3.260 0.00112
## MONTH class1        -18.25823   68.29452     -0.267 0.78920
## MONTH class2       1574.66656  282.80923      5.568 0.00000
## MONTH class3       -479.68502  375.03154     -1.279 0.20088
## 
## 
## Variance-covariance matrix of the random-effects:
##           intercept
## intercept  494489.9
## 
##                                  coef         Se
## Residual standard error:   2316.61529  194.50568
```

### 8.1.4 4-class model

```
summary(model_IPAQ_n4)
```

```
## Heterogenous linear mixed model 
##      fitted by maximum likelihood method 
##  
## hlme(fixed = MET_MIN_WK ~ MONTH, mixture = ~MONTH, random = ~1, 
##     subject = "patient", ng = 4, data = DB_PRED_IPAQ_6_12, var.time = "MONTH")
##  
## Statistical Model: 
##      Dataset: DB_PRED_IPAQ_6_12 
##      Number of subjects: 77 
##      Number of observations: 154 
##      Number of latent classes: 4 
##      Number of parameters: 13  
##  
## Iteration process: 
##      The program stopped abnormally. No results can be displayed.
```

### 8.1.5 5-class model

```
summary(model_IPAQ_n5)
```

```
## Heterogenous linear mixed model 
##      fitted by maximum likelihood method 
##  
## hlme(fixed = MET_MIN_WK ~ MONTH, mixture = ~MONTH, random = ~1, 
##     subject = "patient", ng = 5, data = DB_PRED_IPAQ_6_12, var.time = "MONTH")
##  
## Statistical Model: 
##      Dataset: DB_PRED_IPAQ_6_12 
##      Number of subjects: 77 
##      Number of observations: 154 
##      Number of latent classes: 5 
##      Number of parameters: 16  
##  
## Iteration process: 
##      The program stopped abnormally. No results can be displayed.
```

## 8.2 Compare the models

### 8.2.1 Analyse the metrics of the models

```
tar_read(compa_latent_mixed_models_table_IPAQ)
```

```
##                        AIC          BIC   entropy   %class1  %class2  %class3
## model_IPAQ_n1 2.985761e+03 2.995137e+03 1.0000000 100.00000       NA       NA
## model_IPAQ_n2 2.918285e+03 2.934691e+03 0.9956238  93.50649 6.493506       NA
## model_IPAQ_n3 2.917214e+03 2.940652e+03 0.9379943  88.31169 5.194805 6.493506
## model_IPAQ_n4 2.000000e+09 2.000000e+09 1.0000000   0.00000 0.000000 0.000000
## model_IPAQ_n5 2.000000e+09 2.000000e+09 1.0000000   0.00000 0.000000 0.000000
##               %class4 %class5
## model_IPAQ_n1      NA      NA
## model_IPAQ_n2      NA      NA
## model_IPAQ_n3      NA      NA
## model_IPAQ_n4       0      NA
## model_IPAQ_n5       0       0
```

```
lcmm::summaryplot(
  model_IPAQ_n1,
  model_IPAQ_n2,
  model_IPAQ_n3,
  model_IPAQ_n4,
  model_IPAQ_n5,
  which = c("AIC", "BIC", "entropy")
)
```

### 8.2.2 Posterior classification of the models

#### 8.2.2.1 2-class model

```
lcmm::postprob(model_IPAQ_n2)
```

```
##  
## Posterior classification: 
##   class1 class2
## N  72.00   5.00
## %  93.51   6.49
##  
## Posterior classification table: 
##      --> mean of posterior probabilities in each class 
##        prob1 prob2
## class1 1.000 0.000
## class2 0.012 0.988
##  
## Posterior probabilities above a threshold (%): 
##          class1 class2
## prob>0.7    100    100
## prob>0.8    100    100
## prob>0.9    100    100
##
```

#### 8.2.2.2 3-class model

```
lcmm::postprob(model_IPAQ_n3)
```

```
##  
## Posterior classification: 
##   class1 class2 class3
## N  68.00   4.00   5.00
## %  88.31   5.19   6.49
##  
## Posterior classification table: 
##      --> mean of posterior probabilities in each class 
##         prob1  prob2  prob3
## class1 0.9805 0.0000 0.0195
## class2 0.0000 1.0000 0.0000
## class3 0.1326 0.0111 0.8563
##  
## Posterior probabilities above a threshold (%): 
##          class1 class2 class3
## prob>0.7  97.06    100     80
## prob>0.8  97.06    100     80
## prob>0.9  92.65    100     60
##
```

#### 8.2.2.3 4-class model

```
lcmm::postprob(model_IPAQ_n4)
```

```
##  
## Posterior classification: 
##   class1 class2 class3 class4
## N      0      0      0      0
## %      0      0      0      0
##  
## Posterior classification table: 
##      --> mean of posterior probabilities in each class 
##        prob1 prob2 prob3 prob4
## class1   NaN   NaN   NaN   NaN
## class2   NaN   NaN   NaN   NaN
## class3   NaN   NaN   NaN   NaN
## class4   NaN   NaN   NaN   NaN
##  
## Posterior probabilities above a threshold (%): 
##          class1 class2 class3 class4
## prob>0.7    NaN    NaN    NaN    NaN
## prob>0.8    NaN    NaN    NaN    NaN
## prob>0.9    NaN    NaN    NaN    NaN
##
```

#### 8.2.2.4 5-class model

```
lcmm::postprob(model_IPAQ_n5)
```

```
##  
## Posterior classification: 
##   class1 class2 class3 class4 class5
## N      0      0      0      0      0
## %      0      0      0      0      0
##  
## Posterior classification table: 
##      --> mean of posterior probabilities in each class 
##        prob1 prob2 prob3 prob4 prob5
## class1   NaN   NaN   NaN   NaN   NaN
## class2   NaN   NaN   NaN   NaN   NaN
## class3   NaN   NaN   NaN   NaN   NaN
## class4   NaN   NaN   NaN   NaN   NaN
## class5   NaN   NaN   NaN   NaN   NaN
##  
## Posterior probabilities above a threshold (%): 
##          class1 class2 class3 class4 class5
## prob>0.7    NaN    NaN    NaN    NaN    NaN
## prob>0.8    NaN    NaN    NaN    NaN    NaN
## prob>0.9    NaN    NaN    NaN    NaN    NaN
##
```

**Comment**: The 4-class and the 5-class models did not
converge. The 2-class model seemed to be a reasonable choice.

### 8.2.3 Assess the chosen model

```
plot(model_IPAQ_n2)
```

### 8.2.4 Visualize the fixed effetcs of the model

```
tar_read(plot_preds_IPAQ)
```

## 8.3 Analyse the predictors of the latent classes of the model

```
tar_load(predictors_IPAQ_classes)
summary(predictors_IPAQ_classes)
```

```
## Secondary multinomial model for external class predictor 
##      fitted by maximum likelihood method 
##  
## externVar(model = model_IPAQ_n2, subject = "patient", classmb = ~DIST_6MWT_M0 + 
##     MET_MIN_WK_M0 + MOTIVATION_CLUSTER_M0 + meteo_defavorable + 
##     manque_temps, data = DB_PRED_IPAQ_6_12, method = "twoStageJoint")
##  
## Statistical Model: 
##      Dataset: DB_PRED_IPAQ_6_12 
##      Number of subjects: 76 
##      Number of latent classes: 2 
##      Number of parameters: 6  
##  
## Iteration process: 
##      Convergence criteria satisfied 
##      Number of iterations:  11 
##      Convergence criteria: parameters= 2.2e-07 
##                          : likelihood= 1.3e-08 
##                          : second derivatives= 1.4e-10 
##  
## Goodness-of-fit statistics: 
##      maximum log-likelihood: -1433.2  
##      AIC: 2878.4  
##      BIC: 2892.38  
##  
##  
##  
## Maximum Likelihood Estimates: 
##  
## Fixed effects in the class-membership model:
## (the class of reference is the last class) 
## 
##                                                      coef    Se**   Wald
## intercept class1                                  1.29289 4.05010  0.319
## DIST_6MWT_M0 class1                               0.00047 0.00636  0.074
## MET_MIN_WK_M0 class1                              0.00024 0.00024  1.004
## MOTIVATION_CLUSTER_M0Very High AU-High IR class1  0.02629 0.99719  0.026
## meteo_defavorable1 class1                        -0.30177 0.97011 -0.311
## manque_temps1 class1                              0.52264 1.16166  0.450
##                                                  p-value
## intercept class1                                 0.74956
## DIST_6MWT_M0 class1                              0.94087
## MET_MIN_WK_M0 class1                             0.31554
## MOTIVATION_CLUSTER_M0Very High AU-High IR class1 0.97897
## meteo_defavorable1 class1                        0.75574
## manque_temps1 class1                             0.65278
## 
##  ** total variance estimated through the Hessian of the joint likelihood 
##
```
